# Ketanserin Reverses the Acute Response to LSD in a Randomized, Double-Blind, Placebo-Controlled, Crossover Study in Healthy Participants

**DOI:** 10.1093/ijnp/pyac075

**Published:** 2022-11-07

**Authors:** Anna M Becker, Aaron Klaiber, Friederike Holze, Ioanna Istampoulouoglou, Urs Duthaler, Nimmy Varghese, Anne Eckert, Matthias E Liechti

**Affiliations:** Clinical Pharmacology and Toxicology, Department of Biomedicine and Department of Clinical Research, University Hospital Basel and University of Basel, Basel, Switzerland; Department of Pharmaceutical Sciences, University of Basel, Basel, Switzerland; Clinical Pharmacology and Toxicology, Department of Biomedicine and Department of Clinical Research, University Hospital Basel and University of Basel, Basel, Switzerland; Department of Pharmaceutical Sciences, University of Basel, Basel, Switzerland; Clinical Pharmacology and Toxicology, Department of Biomedicine and Department of Clinical Research, University Hospital Basel and University of Basel, Basel, Switzerland; Department of Pharmaceutical Sciences, University of Basel, Basel, Switzerland; Clinical Pharmacology and Toxicology, Department of Biomedicine and Department of Clinical Research, University Hospital Basel and University of Basel, Basel, Switzerland; Department of Pharmaceutical Sciences, University of Basel, Basel, Switzerland; Clinical Pharmacology and Toxicology, Department of Biomedicine and Department of Clinical Research, University Hospital Basel and University of Basel, Basel, Switzerland; Department of Pharmaceutical Sciences, University of Basel, Basel, Switzerland; Psychiatric University Hospital, University of Basel, Basel, Switzerland; Psychiatric University Hospital, University of Basel, Basel, Switzerland; Transfaculty Research Platform Molecular and Cognitive Neuroscience, University of Basel, Basel, Switzerland; Clinical Pharmacology and Toxicology, Department of Biomedicine and Department of Clinical Research, University Hospital Basel and University of Basel, Basel, Switzerland; Department of Pharmaceutical Sciences, University of Basel, Basel, Switzerland

**Keywords:** LSD, ketanserin, interaction, subjective effects, pharmacokinetics

## Abstract

**Background:**

Lysergic acid diethylamide (LSD) is currently being investigated in psychedelic-assisted therapy. LSD has a long duration of acute action of 8–11 hours. It produces its acute psychedelic effects via stimulation of the serotonin 5-hydroxytryptamine-2A (HT_2A_) receptor. Administration of the 5-HT_2A_ antagonist ketanserin before LSD almost fully blocks the acute subjective response to LSD. However, unclear is whether ketanserin can also reverse the effects of LSD when administered after LSD.

**Methods:**

We used a double-blind, randomized, placebo-controlled, crossover design in 24 healthy participants who underwent two 14-hour sessions and received ketanserin (40 mg p.o.) or placebo 1 hour after LSD (100 µg p.o.). Outcome measures included subjective effects, autonomic effects, acute adverse effects, plasma brain-derived neurotrophic factor levels, and pharmacokinetics up to 12 hours.

**Results:**

Ketanserin reversed the acute response to LSD, thereby significantly reducing the duration of subjective effects from 8.5 hours with placebo to 3.5 hours. Ketanserin also reversed LSD-induced alterations of mind, including visual and acoustic alterations and ego dissolution. Ketanserin reduced adverse cardiovascular effects and mydriasis that were associated with LSD but had no effects on elevations of brain-derived neurotrophic factor levels. Ketanserin did not alter the pharmacokinetics of LSD.

**Conclusions:**

These findings are consistent with an interaction between ketanserin and LSD and the view that LSD produces its psychedelic effects only when occupying 5-HT_2A_ receptors. Ketanserin can effectively be used as a planned or rescue option to shorten and attenuate the LSD experience in humans in research and LSD-assisted therapy.

**Trial registry:**

ClinicalTrials.gov (NCT04558294)

**Significance Statement**

Lysergic acid diethylamide (LSD) is being investigated in psychedelic-assisted therapy. Therapy sessions with LSD are long due to its duration of acute action of 8–11 hours. Ketanserin prevents effects of LSD when given before LSD. However, unknown is whether LSD’s effects can also be blocked once established. Therefore, we tested whether ketanserin (40 mg) does reduce the duration of action of LSD when given 1 hour after LSD (100 μg). In line with our hypothesis, ketanserin effectively reduced the duration of action of LSD from 8.5 to 3.5 hours. We conclude that ketanserin can be useful in LSD-assisted therapy to shorten the acute response to LSD as a planned or emergency treatment and therefore make this form of therapy safer and/or more flexible.

## INTRODUCTION

Psychedelic substances, including lysergic acid diethylamide (LSD) and psilocybin, are investigated as possible treatments to assist psychotherapy (Gasser et al., 2014; Griffiths et al., 2016; Ross et al., 2016; Carhart-Harris et al., 2021; Davis et al., 2021). The subjective and possible therapeutic effects of LSD and psilocybin may be comparable, but the duration of the acute effects of LSD are longer than psilocybin (Holze et al., 2022). Additionally, the acute effects of both substances are mainly positive in clinical settings, but negative experiences, including anxiety, may occur at higher doses (Schmid et al., 2015; Holze et al., 2021b) or in susceptible people. Psilocybin and LSD produce their acute subjective effects in humans via an interaction with the serotonin 5-hydroxytryptamine-2A (5-HT_2A_) receptor (Vollenweider et al., 1998; Madsen et al., 2019; Holze et al., 2021b). Ketanserin potently binds to the 5-HT_2A_ receptor with a binding constant (K_i_) of approximately 3.5 nM (Schmid et al., 2021). Additionally, ketanserin binds to adrenergic α_1A_ and H_1_ histaminergic receptors. Acute side effects include dry mouth and sedation (Brogden and Sorkin, 1990). The subjective effects of classic psychedelics, including LSD and psilocybin, generally can be prevented by the 5-HT_2_ receptor antagonist ketanserin prior to administration of the psychedelic (Vollenweider et al., 1998; Valle et al., 2016; Preller et al., 2017; Holze et al., 2021b). For example, ketanserin (40 mg) administration 1 hour before the administration of LSD doses of 100 µg (Preller et al., 2017) and 200 µg (Holze et al., 2021b) almost completely prevented the acute effects of LSD. However, unclear is whether an LSD experience can also be attenuated or shortened by ketanserin administration after LSD once psychedelic effects have already been established. LSD is a very potent substance that is psychoactive at doses as low as 10 µg (Holze et al., 2021a), with high binding affinity for 5-HT_2A_ receptors (K_i_ of approximately 5 nM). Moreover, LSD shows similarly high affinity for other serotonin receptors, including 5-HT_1A_ and 5-HT_2C_ receptors, as well as for adrenergic α_1A_ and H_1_ histaminergic receptors. LSD also binds with lower affinity to dopamine D_1-3_ receptors (Rickli et al., 2016). Additionally, structural biology studies have shown a strong and unique binding pose of LSD to the 5-HT_2A_ receptor, and this molecular interaction at the binding site could underlie the long duration of action of LSD in humans (Wacker et al., 2017). LSD potentially could be trapped in the receptor pocket in a manner that does not allow antagonism by a receptor antagonist, thus making its downstream effects irreversible. A cascade of intracellular messenger system processes may continue once the receptor is activated and thus might not be influenced by ketanserin when administered after LSD. On the other hand, the time course of the acute subjective action of LSD indicates that it acts only as long as it is present in the body according to its concentration-time curve. Therefore, no special mechanisms at the receptor would be needed to explain its duration of action in humans (Aghajanian and Bing, 1964; Holze et al., 2021b). Instead, its duration of action would be well explained by its pharmacokinetic characteristics. The subjective effects of LSD last an average of approximately 8.5 and approximately 11 hours after the administration of doses of 100 and 200 µg, respectively (Holze et al., 2019; Holze et al., 2021b; Holze et al., 2022), consistent with its plasma half-life of approximately 4 hours (Holze et al., 2021b). Similarly, the time curve of subjective effects of psilocybin and its duration of action of approximately 6 hours are consistent with the plasma concentration-time curve and half-life of its active metabolite psilocin (t_1/2_ = approximately 2.5 hours) (Holze et al., 2022). No 5-HT_2A_ receptor occupancy studies are yet available for LSD in humans, but a positron emission tomography 5-HT_2A_ occupancy study that used psilocybin as a 5-HT_2A_ receptor agonist showed receptor occupancies between 43% and 72%, associated subjective effects (40%–100%), and a positive correlation between plasma psilocin concentrations and subjective effects (Madsen et al., 2019; Madsen and Knudsen, 2021).

Due to LSD’s long duration of action, LSD studies require more resources (e.g., time and personnel) than psilocybin studies (Holze et al., 2022). This is one reason why psilocybin has been used in most recent clinical trials that evaluated the efficacy of psychedelics to assist psychotherapy (Grob et al., 2011; Carhart-Harris et al., 2016; Griffiths et al., 2016; Ross et al., 2016; Carhart-Harris et al., 2018). However, few modern studies of psychedelic-assisted therapy have also used LSD despite its longer duration of action (Gasser et al., 2014; Gasser et al., 2015), and LSD was the most investigated psychedelic in the 1960s to 1970s (Krebs and Johansen, 2012). Remaining to be determined is whether there are differences in therapeutic indications and adverse effects between LSD and psilocybin beyond their duration of action (Holze et al., 2022). Additionally, unknown is whether the duration of action of LSD could potentially be shortened by ketanserin.

Ketanserin could also be a rescue medication in patients who do not tolerate the effects of LSD. Although acute effects of LSD are mostly perceived as positive, it may induce strong feelings of anxiety in some individuals and/or at higher doses (Schmid et al., 2015; Holze et al., 2021b). In the clinical setting, feelings of anxiety are usually transient and can be treated with verbal support by the session supervisor. However, pharmacological treatment may be needed in rare cases, and ketanserin may be an ideal treatment option to antagonize the subjective effects of psychedelics if needed in emergency cases.

The present study investigated whether the acute subjective effects of LSD (100 µg) can be shortened by ketanserin (40 mg) if it is administered 1 hour after LSD administration. The primary predefined hypothesis was that ketanserin would shorten the subjective response, assessed by a visual analog scale (VAS; i.e., “any drug effect” duration), compared with placebo. Secondary predefined hypotheses included overall smaller subjective effects (parametrized as VAS E_max_ and area under the effect curve [AUEC] values), smaller overall alterations of consciousness (total 5 Dimensions of Altered States of Consciousness [5D-ASC] score), and mystical experiences (total Mystical-type Experiences Questionnaire [MEQ30] score), smaller autonomic effects, and no alteration in LSD pharmacokinetics after ketanserin compared with placebo.

## METHODS

### Study Design

The study used a double-blind, placebo-controlled, crossover design with 2 experimental test sessions to investigate the response to LSD (100 µg p.o.) with the consecutive administration of either ketanserin (40 mg p.o.) or placebo 1 hour after LSD administration. The treatment order was random and counterbalanced. Test days were separated by at least 10 days (mean: 23 days), since several previous studies used similar between-session intervals and documented no carry-over or order effects (Holze et al., 2021b; Holze et al., 2022). The study was conducted in accordance with the Declaration of Helsinki and International Conference on Harmonization Guidelines in Good Clinical Practice. The study protocol (including a statistical analysis plan) was approved by the Ethics Committee of Northwest Switzerland (Ethikkommission Nordwest- und Zentralschweiz; Project-ID: 2020-00614) and the Swiss Federal Office for Public Health. The study was registered at ClinicalTrials.gov (NCT04558294). All participants provided written informed consent and were paid for their participation.

### Participants

Twenty-six participants were recruited by word of mouth or by the ClinicalTrials.gov register. One participant dropped out before the first study day, and 1 dropped out after completing the first study day (supplementary Fig. 8). Thus, 24 healthy participants completed the study (12 women, 12 men; 34 ± 12 years old [mean ± SD]; range, 25–64 years). Mean body weight was 71 kg. Four women used a hormonal contraceptive. Drug administration timing did not consider the menstrual cycle for practical reasons. The exclusion criteria were age <25 years or >65 years, pregnancy (urine pregnancy test at screening and before each test session), personal history of major psychiatric disorders (assessed by the Semi-structured Clinical Interview for Diagnostic and Statistical Manual of Mental Disorders 4th edition, Axis I disorders), family (first-degree relative) history of psychotic disorders, the use of medications that may interfere with the study medications (e.g., antidepressants, antipsychotics, and sedatives), chronic or acute physical illness (e.g., abnormal physical exam, electrocardiogram, or hematological and chemical blood analyses), tobacco smoking >10 cigarettes/d, lifetime prevalence of hallucinogenic substance use >20 times or illicit drug use within the last 2 months (except Δ^9^-tetrahydrocannabinol), and during the study period (determined by urine drug tests). The participants were asked to consume no more than 20 standard alcoholic drinks/week and have no more than 1 drink on the day before the test sessions. Eleven participants had experiences with hallucinogenic substances, 3 of whom previously had used LSD (1–2 times).

Additional information on prior substance use is described in the Supplementary Methods online.

### Study Drugs

LSD (D-lysergic acid diethylamide freebase, high-performance liquid chromatography purity >99%; Lipomed AG, Arlesheim, Switzerland) was administered as an oral solution that was produced according to good manufacturing practice in units that contained 100 µg LSD in 1 mL of 96% ethanol (Holze et al., 2019). The exact analytically confirmed LSD freebase content (mean ± SD) was 92.53 ± 1.89 µg (n = 10 samples), consistent with uniformity of dosage units and the target dosage. Stability of the formulation for longer than the study period was documented in an identically produced previous batch (Holze et al., 2019). Ketanserin was obtained as the marketed drug Ketensin (20 mg, Janssen-Cilag, Leiden, the Netherlands) and encapsulated with opaque capsules to ensure blinding. Placebo consisted of identical opaque capsules filled with mannitol. A double-dummy method was used. At the end of each session and at the end of the study, the participants were asked to retrospectively guess their treatment assignment.

### Study Procedures

The study included a screening visit, two 14-hour test sessions, and an end-of-study visit. The sessions were conducted in a calm hospital room. Only 1 research participant and 1 investigator were present during each test session. The test sessions began at 7:30 am. A urine sample was taken to verify abstinence from drugs of abuse, and a urine pregnancy test was performed in women. The participants then underwent baseline measurements. LSD was administered at 9:00 am, and ketanserin or placebo was administered at 10:00 am. Outcome measures were assessed for 10 hours after LSD administration. Standardized lunches and dinners were served at approximately 1:30 pm and 6:00 pm, respectively. The participants were never alone during the test sessions and were sent home at 9:30 pm in the company of another person.

### Subjective Drug Effects

Subjective effects over time were repeatedly assessed using VASs (Schmid et al., 2015; Holze et al., 2020, 2021b, 2022) before (0 hours) and 0.5, 1, 1.5, 2, 2.5, 3, 3.5, 4, 5, 6, 7, 8, 9, 10, 11, and 12 hours after LSD administration. The time to effect onset, time to maximal effect, time to effect offset, and effect duration were assessed using “any drug effect” VAS effect-time plots and an onset/offset threshold of 10% of the maximum individual response as previously described (Holze et al., 2019, 2021b). The Adjective Mood Rating Scale (Janke and Debus, 1978) was used before (0 hours) and 3, 6, 9, and 12 hours after LSD administration. To retrospectively rate overall psychedelic alterations, the 5D-ASC (Studerus et al., 2010) was administered 12 hours after LSD administration. The questionnaire contains 94 items that are rated on VASs and grouped into 5 dimensions. The 3 main dimensions are “oceanic boundlessness,” “anxious ego-dissolution,” and “visionary restructuralization,” and their total (3D-OAV score) can be used as a measure of the overall intensity of psychedelic-specific alterations of mind in addition to the 5D-ASC total score (Liechti et al., 2017). Mystical experiences were assessed 12 hours after LSD administration using the States of Consciousness Questionnaire (Griffiths et al., 2006; Liechti et al., 2017) that includes the 30-item MEQ30 (Barrett et al., 2015).

Subjective effect measurements are described in detail in the supplementary Methods online.

### Autonomic and Adverse Effects

Blood pressure, heart rate, tympanic body temperature, and pupil size were repeatedly measured (Hysek and Liechti, 2012; Schmid et al., 2015). Adverse effects were assessed 1 hour before and 12 hours after LSD administration using the List of Complaints (Zerssen, 1976). Adverse events that occurred outside the test sessions were recorded at the beginning of the next test session and at the end of the study visit.

### Plasma Brain-Derived Neurotrophic Factor (BDNF) Levels

Plasma BDNF levels were measured at baseline (0 hours) and 6, 9, and 12 hours after LSD administration as previously described (Akimoto et al., 2019; Holze et al., 2020, 2021b; Hutten et al., 2020).

### Plasma LSD Concentrations

Blood was collected into lithium heparin tubes. The blood samples were immediately centrifuged, and plasma was subsequently stored at −80°C until analysis. Plasma concentrations of LSD were determined by ultra-high-performance liquid chromatography tandem mass spectrometry with a lower limit of quantification of 10 pg/mL (Holze et al., 2019). Plasma ketanserin concentrations were also determined using liquid chromatography tandem mass spectrometry. Additional information on the bioanalysis of ketanserin is described in the supplementary Methods online. Pharmacokinetic parameters were estimated using non-compartmental methods in Phoenix WinNonlin 8.3 (Certara, Princeton, NJ, USA) as previously described (Holze et al., 2019).

### Data and Statistical Analysis

The primary study endpoint was the duration of the subjective response as assessed with the VAS “any drug effect.” The onset, t_max_, offset, and effect duration were defined in the any drug effect-time plots using a threshold of 10% of the maximum individual response using Phoenix WinNonlin 6.4 and as previously described (Holze et al., 2019, 2021b). Peak (E_max_ and/or E_min_), peak change from baseline (ΔE_max_), and AUEC values were determined for repeated measures and were compared as additional endpoints. The values were analyzed using paired 2-sided *t* tests. The data were analyzed using RStudio 1.3.1103 software (RStudio, PBC, Boston, MA, USA). The criterion for significance was *P* < .05. Sex and body weight were not taken into account because previous studies have not reported any differences (Dolder et al., 2017; Holze et al., 2019, 2021b). No correction for multiple testing was used based on the priori definition of a limited set of outcomes with specific hypotheses. A priori power analysis estimated sufficient power for the primary endpoint with a sample size >16. A sample size of 24 accounted for the secondary endpoints, although these analyses were more exploratory and/or confirmatory of the primary endpoint findings using alternative measures. Additional information on sample size calculation is described in detail in the supplementary Methods online. The data and statistical analysis comply with the recommendations on experimental design and analysis in pharmacology.

## RESULTS

### Subjective Effects

**Figure 1.**
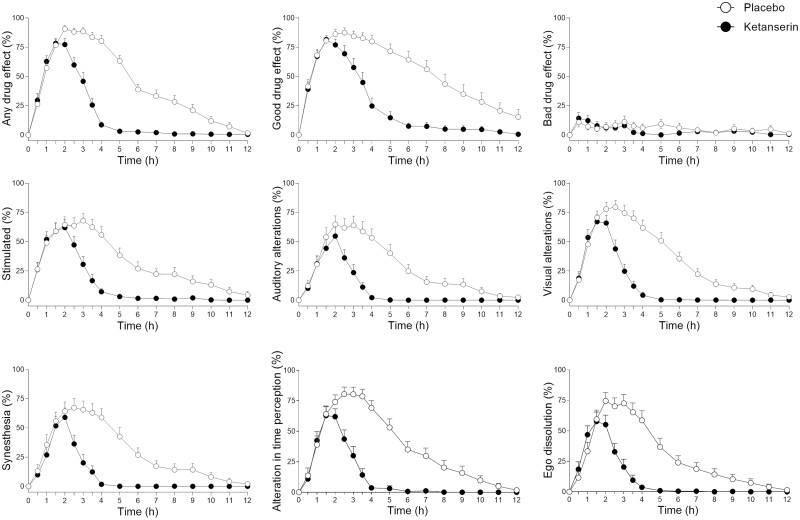
Acute subjective effects of lysergic acid diethylamide (LSD) over time on visual analog scales (VASs). Ketanserin reversed LSD-induced subjective effects compared with placebo. LSD was administered at t = 0 hours. Ketanserin or placebo was administered at t = 1 hour. The data are expressed as the mean ± SEM in 24 participants (12 men, 12 women). Additional subjective effects are shown in supplementary Figure 1. The corresponding maximal effect and area under the effect curve (AUEC) values and statistics are shown in Table 2.

**Figure 2.**
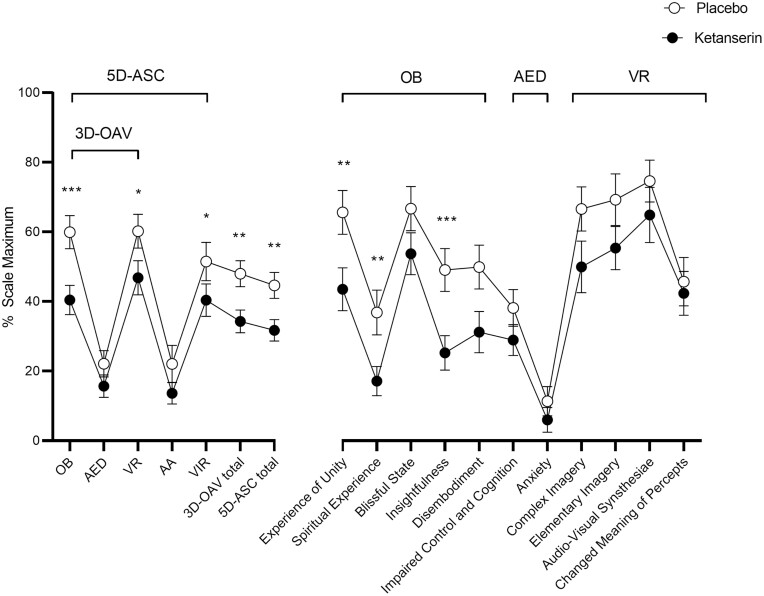
Lysergic acid diethylamide (LSD) effects on 5 Dimensions of Altered States of Consciousness (5D-ASC) scale. Ketanserin administration 1 hour after LSD significantly reduced LSD-typical alterations of mind compared with placebo, indicated mainly by significantly lower three dimension (3D-OAV) and five dimension (5D-ASC) total scores. Abbreviations: AA, auditory alterations; AED, anxious ego-dissolution; OB, oceanic boundlessness; VIR, vigilance reduction; VR, visionary restructuralization. The data are expressed as the mean ± SEM percentage of maximally possible scale scores in 24 participants (12 men, 12 women). *P < .05, ** *P < *.01, *** *P* < .001, significant difference between ketanserin and placebo. Full statistics are shown in supplementary Table 2.

Ketanserin significantly reduced the “any drug effect” duration of LSD from an average of 8.5 hours with placebo to 3.5 hours (Figure 1; Table 1). Individual data plots are shown in supplementary Fig. 7. As expected, the maximal effect of LSD was not significantly reduced by ketanserin, but significant mean reductions were observed already at 2 hours and thereafter (Figure 1; Table 1). Ketanserin also reversed LSD-induced increases in VAS ratings of “good drug effects,” “stimulated,” “auditory alterations,” “visual alterations,” “synesthesia,” “alterations in time perception,” and “ego-dissolution” (Figure 1). The significance of these effects was indicated by a lower AUEC for LSD and ketanserin compared with LSD and placebo (Table 2). Specifically, reductions of AUEC values by 60%–70% were observed for “any drug effects,” typical LSD effects (e.g., ego dissolution, visual, auditory, and time perception alterations), and nausea (Figure 1; Table 2). Ketanserin also reduced “bad drug effect” ratings after LSD, but the moderating effect was not significant because only minimal/few bad drug effects occurred at the dose of LSD used (Figure 2; Table 2). Ketanserin significantly reversed LSD-typical alterations in “introversion,” “emotional excitation,” and reductions of “concentration” on the Adjective Mood Rating Scale (supplementary Fig. 2; supplementary Table 1). Moreover, it significantly reduced LSD-typical alterations of mind on the 5D-ASC questionnaire by reducing the 3D-OAV and 5D-ASC total scores compared with placebo (Figure 2; supplementary Table 2). However, ketanserin did not significantly alter overall mystical experiences, as indicated by the MEQ30 total score, that were produced by LSD (supplementary Fig. 3; supplementary Table 2).

**Table 1.** Parameters Characterizing the Subjective LSD Drug Effect-Time Curve

Parameter	Ketanserin	Placebo	t_23_	*P*
Time to onset (h)	0.3 ± 0.2	0.3 ± 0.2	−0.1	.955
	(0.1–0.7)	(0.1–0.7)		
Time to offset (h)	3.8 ± 1.3	8.6 ± 2.0	−13.4	.000***
	(2.1–7.0)	(5.0–11.5)		
Time to maximal effect (h)	1.9 ± 0.7	2.0 ± 0.9	−0.1	.922
	(1.0–3.0)	(0.5–4.0)		
Effect duration (h)	3.5 ± 1.3	8.5 ± 2.2	−9.9	.001**
	(1.6–9.6)	(5.3–12.0)		
Maximal effect (%)	89 ± 13	93 ± 12	−1.6	.118
	(52–100)	(60–100)		
Effect at 2 h (%)	77 ± 25	91 ± 15	−2.6	.015*
	(11–100)	(47–100)		
Effect at 4 h (%)	9 ± 12	80 ± 23	−12.5	.000***
	(0–34)	(18–100)		
Effect at 6 h (%)	3 ± 5	39 ± 21	−8.5	.000***
	(0–15)	(2–69)		
Effect at 8 h (%)	1 ± 2	28 ± 28	−5.0	.000***
	(0–9)	(0–86)		
AUEC (h*pg/mL)	202 ± 79	513 ± 169	−10.2	.001**
	(69–322)	(194–958)		

Abbreviations: AUEC, area under the effect curve; LSD = lysergic acid diethylamide.

*P < .05, **P < .01, ***P < .001; n = 24. Values are mean ± SD (range).

**Table 2.** Mean Values and Statistics for the Acute Effects of LSD on the VAS

Effect		Ketanserin	Placebo	t_23_	*P*
		mean ± SEM	mean ± SEM		
Any drug effect	ΔE_max_	89 ± 3	93 ± 2	−1.6	.118
	AUEC	202 ± 16	513 ± 35	−10.2	.001**
Good drug effect	ΔE_max_	92 ± 3	94 ± 2	−1.0	.320
	AUEC	280 ± 28	647 ± 54	−7.5	.000***
Bad drug effect	ΔE_max_	21 ± 5	20 ± 6	0.1	.955
	AUEC	41 ± 20	67 ± 27	−1.8	.083
Stimulated	ΔE_max_	71 ± 6	77 ± 6	−0.9	.356
	AUEC	158 ± 20	381 ± 45	−5.2	.000***
Fear	ΔE_max_	9 ± 5	9 ± 4	−0.0	.963
	AUEC	16 ± 12	43 ± 31	−1.4	.175
Ego dissolution	ΔE_max_	67 ± 7	81 ± 6	−2.8	.010*
	AUEC	122 ± 18	347 ± 46	−6.1	.000***
Nausea	ΔE_max_	22 ± 6	28 ± 6	−1.3	.208
	AUEC	38 ± 18	93 ± 39	2.3	.028*
Tiredness	ΔE_max_	52 ± 6	58 ± 6	−1.4	.175
	AUEC	220 ± 49	345 ± 65	−2.4	.027*
Visual alterations	ΔE_max_	78 ± 5	86 ± 4	−2.6	.016*
	AUEC	143 ± 16	407 ± 42	−7.3	.000***
Auditory alterations	ΔE_max_	65 ± 8	75 ± 7	−2.1	.051
	AUEC	105 ± 15	327 ± 50	−5.3	.000***
Synesthesia	ΔE_max_	74 ± 7	77 ± 7	−0.6	.584
	AUEC	107 ± 14	348 ± 50	−5.7	.000***
Alteration in time perception	ΔE_max_	79 ± 6	86 ± 5	−1.9	.068
	AUEC	137 ± 16	431 ± 46	−7.3	.000***
Insight	ΔE_max_	45 ± 7	59 ± 8	−2.0	.059
	AUEC	71 ± 13	247 ± 46	−4.8	.000***
Talkative	ΔE_max_	8 ± 2	9 ± 3	−0.6	.553
	ΔE_min_	−30 ± 4	−33 ± 4	1.1	.266
	AUEC	524 ± 14	451 ± 31	2.8	.011*
Open	ΔE_max_	14 ± 3	20 ± 4	−1.8	.087
	ΔE_min_	−15 ± 4	−18 ± 4	0.7	.467
	AUEC	608 ± 18	587 ± 39	0.7	.486
Trust	ΔE_max_	25 ± 4	29 ± 4	−1.8	.085
	ΔE_min_	−5 ± 3	−5 ± 3	−0.2	.873
	AUEC	697 ± 38	738 ± 45	−1.8	.082

Abbreviations: AUEC = area under the effect curve; ΔE_max_ = maximal difference from baseline; LSD = lysergic acid diethylamide; SEM = standard error of the mean; VAS = visual analog scale. *P < .05, **P < .01, ***P < .001; n = 24.

### Autonomic and Adverse Effects

Autonomic effects over time and respective peak effects are shown in supplementary Figure 4 and Table 3. Ketanserin significantly reversed LSD-induced elevations of blood pressure and rate pressure product overall (i.e., reductions of AUEC) but not peak responses. Ketanserin also reversed LSD-induced mydriasis (supplementary Fig. 5; supplementary Table 3). Ketanserin did not significantly alter acute adverse effects of LSD on the List of Complaints compared with placebo (supplementary Table 3). The most commonly reported adverse effects of LSD within 12 hours were fatigue (14 participants after ketanserin and 15 participants after placebo), lack of concentration (13 participants after ketanserin and 14 after placebo), and lack of energy (13 participants after ketanserin and 13 after placebo). However, fatigue and lack of energy were more precisely measured by VAS ratings of “tiredness,” which showed a significant overall reduction in the ketanserin condition (Table 2).

### Effects on BDNF

LSD significantly increased peak plasma BDNF levels in the ketanserin and placebo condition compared with baseline. Ketanserin did not influence the LSD-induced increase in BDNF (supplementary Fig. 6; supplementary Table 4).

### Pharmacokinetics

**Figure 3.**
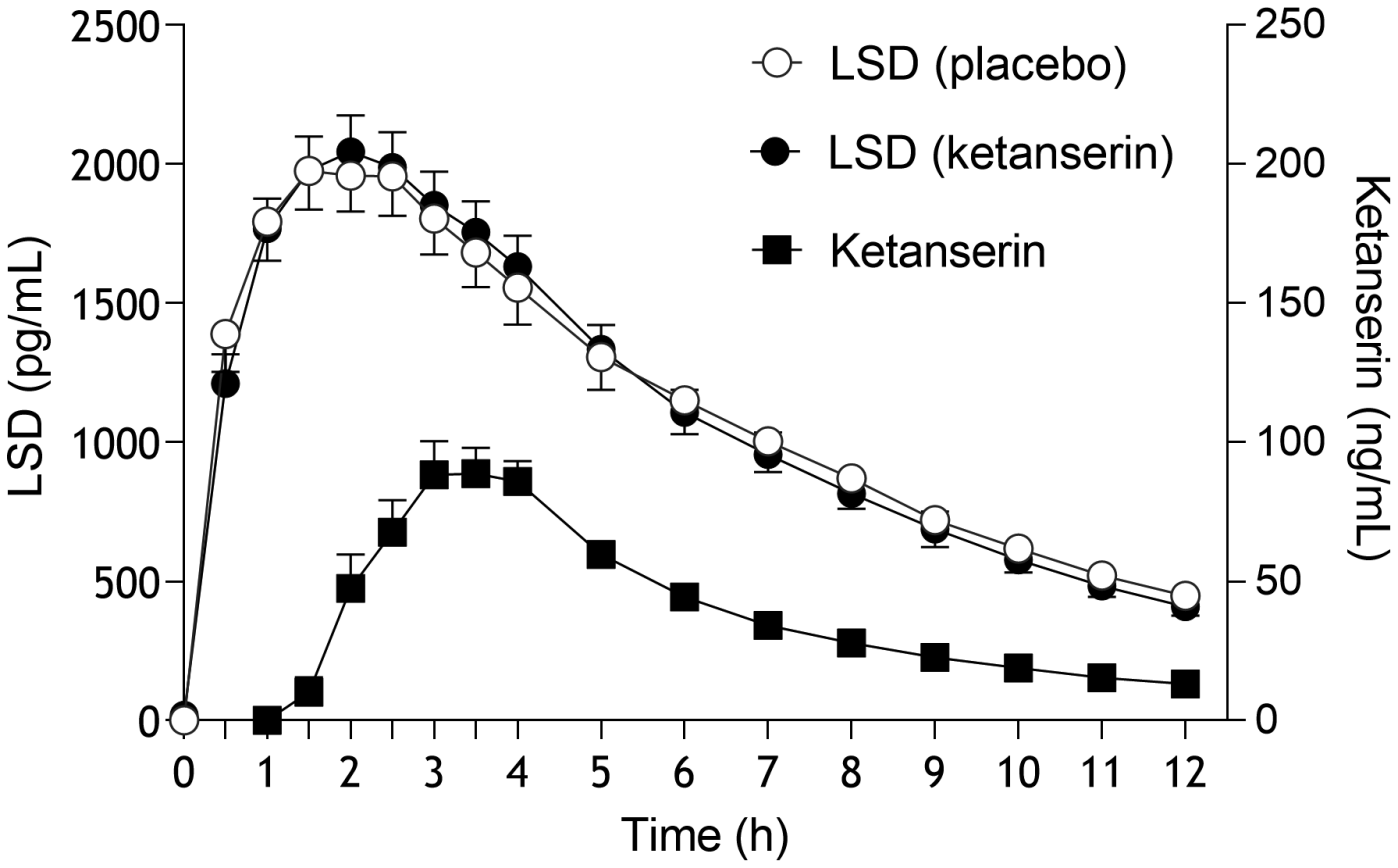
Pharmacokinetics of lysergic acid diethylamide (LSD) and ketanserin. Ketanserin had no effect on the pharmacokinetics of LSD compared with placebo. The data are expressed as the mean ± SEM in 24 participants (12 men, 12 women). LSD was administered at t = 0 hours. Ketanserin or placebo was administered at t = 1 hour. The corresponding pharmacokinetic parameters are listed in Table 3.

Pharmacokinetic parameters are listed in Table 3, and plasma-time curves are shown in Figure 3. Plasma concentrations of LSD and 2-oxo-3-hydroxy LSD were quantified before and up to 12 hours after administration. Ketanserin did not alter the pharmacokinetics of LSD or 2-oxo-3-hydroxy LSD (Table 3). Maximal plasma concentrations of LSD were reached after a mean time of 2 hours in both conditions (Table 3). The terminal elimination half-life of LSD was approximately 4 hours in both conditions (Table 3). Plasma ketanserin levels reached a maximum after a median time of 2 hours (range, 1–5 hours) and declined with a half-life of 3.5 hours.

**Table 3.** Pharmacokinetic Parameters

Metabolite	C_max_ (ng/mL)	t_max_ (h)	t_1/2_ (h)	AUC_12_ (ng·h/mL)	AUC_∞_ (ng·h/mL)	CL/F (L/h)	V_z_/F (L)
LSD and placebo							
LSD	2.13 ± 0.72	2.0 ± 0.8	4.2 ± 1.6	13.8 ± 5.9	17 ± 9	7.2 ± 2.9	39 ± 10
	(1.27–4.26)	(0.5–3.5)	(2.6–8.9)	(6.7–30.8)	(7–43)	(2.3–14.2)	(20–52)
O-H-LSD	0.15 ± 0.44	5.5 ± 1.8	8.3 ± 3.1	1.3 ± 0.4	2.4 ± 0.8	47.4 ± 20.9	532 ± 219
	(0.06–0.24)	(2.5–9.0)	(4.1–14.9)	(0.6–2.2)	(0.8–3.6)	(27.4–123.5)	(231–1211)
LSD and ketanserin							
LSD	2.18 ± 0.63	2.0 ± 0.8	4.1 ± 1.1	13.7 ± 4.3	16.3 ± 6.1	7.0 ± 2.7	39 ± 11
	(1.17–3.19)	(1.0–4.0)	(2.9–7.0)	(7.0–20.7)	(8.1–29.5)	(3.4–12.3)	(24–65)
O-H-LSD	0.15 ± 0.04	4.7 ± 1.5	9.0 ± 3.5	1.3 ± 0.3	2.5 ± 0.8	45.2 ± 16.8	549 ± 172
	(0.08–0.23)	(3.0–9.0)	(5.5–20.9)	(0.7–2.1)	(1.0–4.3)	(23.1–101.5)	(262–964)
Ketanserin	129 ± 46	2.3 ± 1.0	3.5 ± 0.6	442 ± 140	509 ± 157	86 ± 28	442 ± 198
	(44–203)	(1.0–5.0)	(2.5–4.9)	(184–753)	(245–893)	(45–163)	(254–1145)

Abbreviations: AUC = area under the plasma concentration-time curve; AUC_∞_ = AUC from time zero to infinity; AUC_12_ = from time 0-12 h; CL/F = apparent total clearance; C_max_ =  maximum observed plasma concentration; O-H-LSD = 2-oxo-3-hydroxy LSD; T_1/2_ = plasma half-life; T_max_ = time to reach C_max_; V_z_/F = apparent volume of distribution. n = 24.

Values are mean ± SD (range).

## DISCUSSION

The present study demonstrated that the 5-HT_2A_ receptor antagonist ketanserin reversed the subjective and autonomic responses to the prototypical psychedelic LSD in humans. Importantly, this was the case when ketanserin was administered after the effects of LSD had already developed. Specifically, ketanserin (40 mg) given orally 1 hour after the administration of an oral dose of LSD (100 µg) rapidly and markedly reversed LSD’s effects within 2.5 hours of ketanserin administration. Ketanserin reduced the effect duration of LSD from an average of 8.5 hours to 3.5 hours (approximately 60%). The participants mostly reported a rapid normalization of their state of mind (supplementary Fig. 7). Ketanserin did not relevantly reduce the peak response to LSD but had a marked effect on the duration and overall response to LSD over time, statistically confirmed by 60%–70% reductions of typical effects of LSD on VAS AUEC values (Table 2). Ketanserin significantly reduced most aspects of the LSD AUEC response on the VAS, whereas there were smaller or no effects of ketanserin on the 5D-ASC and MEQ. In the present study, ketanserin significantly reduced effects of LSD on the 5D-ASC but not on the MEQ30 total score. It is possible that the 5D-ASC total score is mainly a measure of the overall response of LSD (similar to the AUEC in the VAS), whereas the MEQ total score mainly assesses the peak response to LSD (similar to the E_max_ in the VAS). The differences are unlikely to reflect selective effects of ketanserin on the response to LSD. In fact, ketanserin pretreatment similarly prevented all aspects of the LSD response in humans (Holze et al., 2021b).

Psilocybin is the most commonly investigated psychedelic in psychiatric research. It is currently preferred over LSD, partly because of its shorter duration of action. The duration of action of 20–25 mg psilocybin, a dose equivalent to the 100-µg dose of LSD used in the present study (Holze et al., 2022), is an average of 5.5–6 hours (Griffiths et al., 2016; Becker et al., 2022; Holze et al., 2022) compared with 8.5 hours for LSD. The present data demonstrate that acute subjective LSD effects can be shortened to 3.5 hours when ketanserin is administered 1 hour after LSD administration. Likely, the duration of action could also be adjusted to 4.5 or 5.5 hours when ketanserin is administered 2 or 3 hours after LSD, respectively. These and regimens including higher doses of LSD remain to be confirmed. The present data suggest the possibility of full reversal of the response to LSD at essentially any time and within 2.5 hours after oral ketanserin administration. The intravenous administration of ketanserin would result in an even faster reversal. Additionally, a recent study showed no relevant differences in tolerability or quality of subjective effects of psilocybin and LSD (Holze et al., 2022). In conjunction with the present data, it can be assumed that the time course and effects of psilocybin (20–25 mg) could likely be mimicked by LSD (100 µg) combined with oral ketanserin administration (40 mg) 2–3 hours after LSD administration. Full psychedelic effects on the 5D-ASC and MEQ30 can likely be induced by LSD if ketanserin is administered at 2–3 hours rather than 1 hour after LSD administration.

Ketanserin has known adverse effects, including sedation, hypotension, and nasal congestion (Koudas et al., 2009). However, in the present study, only 2 participants reported nasal congestion after ketanserin administration. Moreover, ketanserin significantly reduced tiredness (Table 2) and concentration problems (supplementary Table 1) associated with LSD. Thus, ketanserin’s antagonistic effects on LSD’s actions seemed to be more relevant than its sedating properties. We cannot fully determine the extent to which hypotensive effects that were observed compared with placebo resulted from ketanserin administration alone or resulted from its antagonism of the blood pressure elevation by LSD. However, blood pressure 5 hours after ketanserin administration was lower than at the start and end of the test sessions, indicating an effect of ketanserin on blood pressure in healthy individuals. Ketanserin also transiently lowered blood pressure for 3 hours when given before LSD and until LSD’s effect started in healthy individuals (Holze et al., 2021b).

Psychedelics induce neuroplastogenic effects and increase markers of neuroregeneration, including BDNF (Ly et al., 2018; Hutten et al., 2020; Dong et al., 2021; Holze et al., 2021b). LSD increased BDNF levels in the present study compared with baseline values, consistent with previous studies (Hutten et al., 2020; Holze et al., 2021b), although the present study did not include a placebo condition for LSD. Interestingly, ketanserin did not reduce LSD-induced elevations of BDNF when administered after LSD in the present study or when administered before LSD in a previous study (Holze et al., 2021b). This finding indicates that LSD may induce BDNF release via mechanisms independent of 5HT_2A_ receptor stimulation and is consistent with the view that distinct mechanisms may mediate the acute mind-altering and neuroregenerative effects of psychedelics (Cao et al., 2022). It remains to be investigated whether the subjective effects of LSD or other psychedelics could be blocked by ketanserin while potentially retaining long-term therapeutic effects. Supporting this possibility, antidepressant-like behavioral and synaptic actions of psilocybin in mice remained intact after treatment with ketanserin (Hesselgrave et al., 2021). However, remaining unknown is whether LSD fully retains any therapeutic effects (if present) in humans if the psychedelic response is blocked or shortened with ketanserin.

The present study also described the pharmacokinetics of an oral LSD solution in healthy individuals. The parameters were consistent with previous studies that used the same formulation (Holze et al., 2019, 2021b, 2022) and were not influenced by ketanserin. Ketanserin elimination kinetics have been described by a 3-compartment model with sequential half-lives of 0.13 hours (t_½*α*_), 2 hours (t_½β_), and 14.3 hours (t_½γ_) (Heykants et al., 1986; Persson et al., 1991). The apparent half-life of ketanserin in the present study is consistent with the t_½β_, which mainly defines elimination within 1–12 hours after administration, whereas t_½γ_ reflects the terminal half-life >24 hours after administration (Heykants et al., 1986; Persson et al., 1991). Despite the slightly shorter half-life of ketanserin compared with LSD and the relatively rapid decrease in plasma ketanserin concentration, ketanserin reduced the response to LSD until the end of the 12-hour session with no apparent rebound. However, single-dose ketanserin administration (40 mg) in the present study may not be sufficient to fully prevent rebound effects of LSD if a higher dose of LSD is used (e.g., 200 µg). Thus, a higher dose of ketanserin or an add-on dose of 20 mg 6–8 hours after the administration of higher doses of LSD may be needed. This remains to be investigated.

The fact that ketanserin persistently blocked the LSD response despite its shorter half-life is notable. Ketanserin is typically used in preclinical research in competitive binding assays or to antagonize effects of psychedelics. Assuming that ketanserin competitively displaces LSD at the receptor, similar or higher effect-site concentrations as LSD would be needed. Plasma ketanserin concentrations reached a maximum of 129 ng/mL (327 nM) within 2.3 hours, consistent with its rapid absorption (Persson et al., 1991). Ketanserin binds to plasma proteins and other tissues. The free fraction in plasma is approximately 5% (Persson et al., 1991). Thus, free peak plasma concentrations of ketanserin were in the range of 16 nM, and free brain concentrations can typically be expected to be in a comparable range. Thus, free peak concentrations of ketanserin at the effect site were likely approximately fourfold higher than its K_i_ value at the 5-HT_2A_ receptor, consistent with the observed complete and sustained antagonism of the LSD response. In contrast, predicted plasma concentrations of LSD that produced half-maximal effects (EC_50_ values) were approximately 1 ng/mL (Dolder et al., 2017; Holze et al., 2019), which is approximately twofold below the peak plasma concentration of LSD that was reached in the present study. These estimations indicate that we used a relatively high dose of ketanserin relative to LSD that was able to fully or almost fully displace LSD from its site of action despite its continued presence in the body. Additionally, the same dose of 40 mg ketanserin given 1 hour before LSD effectively prevented subjective effects of a higher 200-µg dose of LSD (Holze et al., 2021b). Positron emission tomography studies are required to better elucidate 5-HT_2A_ receptor occupancy by both ketanserin and LSD in humans and confirm the above estimations.

The present study has several strengths. First, we used a blinded, placebo-controlled, randomized, balanced design. The crossover design allowed well-powered within-participant comparisons. Second, the pharmaceutical formulation of LSD was produced according to Good Manufacturing Practices, including content definition and stability.

The present study also has limitations. First, the study design did not include a placebo condition for LSD. However, the study validly tested the effects of ketanserin compared with placebo on the non-blinded acute subjective LSD effects reflecting the clinical situation in patients in LSD-assisted treatment where LSD is administered open-label. The acute effects of the dose and formulation of LSD used in the present study have repeatedly been described in previous placebo-controlled studies (Holze et al., 2019; Holze et al., 2020; Holze et al., 2021b; Holze et al., 2022). Second, the study included only healthy participants and only 1 moderately high dose of LSD. Greater and more negative effects may occur in patients and with higher doses while the acute effects of LSD are generally similar in healthy individuals and patients (Liechti et al., 2017; Schmid et al., 2021). Lastly, ketanserin was administered 1 hour after LSD administration to maximize its effects on the LSD response. In practice, the need to antagonize the LSD response may occur several hours after LSD administration, and the effects and potential benefits of ketanserin may be smaller when used later. However, together with findings of trials that tested ketanserin administration prior to LSD, including higher LSD doses (Preller et al., 2017; Holze et al., 2021b), the results indicate the high efficacy of ketanserin likely at any time during the expected LSD response.

In conclusion, the present study supports the view that LSD produces its subjective and autonomic effects in humans predominantly via a primary interaction with the 5-HT_2A_ receptor. Ketanserin can be useful when given after LSD to shorten the acute response to LSD, either as a planned administration or as a rescue treatment in patients who experience a negative acute response. Ketanserin may also be a useful emergency treatment option as a backup for professionals who offer psychedelic-assisted therapy.

## Supplementary Material

pyac075_suppl_Supplementary_Material

## Interest Statement

M.E.L. is a consultant for Mind Medicine, Inc. The other authors have nothing to disclose.

## References

[CIT0001] Aghajanian GK , Bing OH (1964) Persistence of lysergic acid diethylamide in the plasma of human subjects. Clin Pharmacol Ther 5:611–614. 14209776 10.1002/cpt196455611

[CIT0002] Akimoto H , Oshima S, Sugiyama T, Negishi A, Nemoto T, Kobayashi D (2019) Changes in brain metabolites related to stress resilience: metabolomic analysis of the hippocampus in a rat model of depression. Behav Brain Res 359:342–352. 30447240 10.1016/j.bbr.2018.11.017

[CIT0003] Barrett FS , Johnson MW, Griffiths RR (2015) Validation of the revised Mystical Experience Questionnaire in experimental sessions with psilocybin. J Psychopharmacol 29:1182–1190. 26442957 10.1177/0269881115609019 PMC5203697

[CIT0004] Becker AM , Holze F, Grandinetti T, Klaiber A, Toedtli VE, Kolaczynska KE, Duthaler U, Varghese N, Eckert A, Grunblatt E, Liechti ME (2022) Acute effects of psilocybin after escitalopram or placebo pretreatment in a randomized, double-blind, placebo-controlled, crossover study in healthy subjects. Clin Pharmacol Ther 111:886–895. 34743319 10.1002/cpt.2487 PMC9299061

[CIT0005] Brogden RN , Sorkin EM (1990) Ketanserin. A review of its pharmacodynamic and pharmacokinetic properties, and therapeutic potential in hypertension and peripheral vascular disease. Drugs 40:903–949. 2079001 10.2165/00003495-199040060-00010

[CIT0006] Cao D , Yu J, Wang H, Luo Z, Liu X, He L, Qi J, Fan L, Tang L, Chen Z, Li J, Cheng J, Wang S (2022) Structure-based discovery of nonhallucinogenic psychedelic analogs. Science 375:403–411. 35084960 10.1126/science.abl8615

[CIT0007] Carhart-Harris RL , Bolstridge M, Rucker J, Day CM, Erritzoe D, Kaelen M, Bloomfield M, Rickard JA, Forbes B, Feilding A, Taylor D, Pilling S, Curran VH, Nutt DJ (2016) Psilocybin with psychological support for treatment-resistant depression: an open-label feasibility study. Lancet Psychiatry 3:619–627. 27210031 10.1016/S2215-0366(16)30065-7

[CIT0008] Carhart-Harris RL , Bolstridge M, Day CMJ, Rucker J, Watts R, Erritzoe DE, Kaelen M, Giribaldi B, Bloomfield M, Pilling S, Rickard JA, Forbes B, Feilding A, Taylor D, Curran HV, Nutt DJ (2018) Psilocybin with psychological support for treatment-resistant depression: six-month follow-up. Psychopharmacology (Berl) 235:399–408. 29119217 10.1007/s00213-017-4771-x PMC5813086

[CIT0009] Carhart-Harris R , Giribaldi B, Watts R, Baker-Jones M, Murphy-Beiner A, Murphy R, Martell J, Blemings A, Erritzoe D, Nutt DJ (2021) Trial of psilocybin versus escitalopram for depression. N Engl J Med 384:1402–1411. 33852780 10.1056/NEJMoa2032994

[CIT0010] Davis AK , Barrett FS, May DG, Cosimano MP, Sepeda ND, Johnson MW, Finan PH, Griffiths RR (2021) Effects of psilocybin-assisted therapy on major depressive disorder: a randomized clinical trial. JAMA Psychiatry 78:481–489. 33146667 10.1001/jamapsychiatry.2020.3285 PMC7643046

[CIT0011] Dolder PC , Schmid Y, Steuer AE, Kraemer T, Rentsch KM, Hammann F, Liechti ME (2017) Pharmacokinetics and pharmacodynamics of lysergic acid diethylamide in healthy subjects. Clin Pharmacokinetics 56:1219–1230. 10.1007/s40262-017-0513-9 PMC5591798 28197931

[CIT0012] Dong C , Ly C, Dunlap LE, Vargas MV, Sun J, Hwang IW, Azinfar A, Oh WC, Wetsel WC, Olson DE, Tian L (2021) Psychedelic-inspired drug discovery using an engineered biosensor. Cell 184:2779–2792.e18. 33915107 10.1016/j.cell.2021.03.043 PMC8122087

[CIT0013] Gasser P , Holstein D, Michel Y, Doblin R, Yazar-Klosinski B, Passie T, Brenneisen R (2014) Safety and efficacy of lysergic acid diethylamide-assisted psychotherapy for anxiety associated with life-threatening diseases. J Nerv Ment Dis 202:513–520. 24594678 10.1097/NMD.0000000000000113 PMC4086777

[CIT0014] Gasser P , Kirchner K, Passie T (2015) LSD-assisted psychotherapy for anxiety associated with a life-threatening disease: a qualitative study of acute and sustained subjective effects. J Psychopharmacol 29:57–68. 25389218 10.1177/0269881114555249

[CIT0015] Griffiths RR , Richards WA, McCann U, Jesse R (2006) Psilocybin can occasion mystical-type experiences having substantial and sustained personal meaning and spiritual significance. Psychopharmacology (Berl) 187:268–283; discussion 284–292. 16826400 10.1007/s00213-006-0457-5

[CIT0016] Griffiths RR , Johnson MW, Carducci MA, Umbricht A, Richards WA, Richards BD, Cosimano MP, Klinedinst MA (2016) Psilocybin produces substantial and sustained decreases in depression and anxiety in patients with life-threatening cancer: a randomized double-blind trial. J Psychopharmacol 30:1181–1197. 27909165 10.1177/0269881116675513 PMC5367557

[CIT0017] Grob CS , Danforth AL, Chopra GS, Hagerty M, McKay CR, Halberstadt AL, Greer GR (2011) Pilot study of psilocybin treatment for anxiety in patients with advanced-stage cancer. Arch Gen Psychiatry 68:71–78. 20819978 10.1001/archgenpsychiatry.2010.116

[CIT0018] Hesselgrave N , Troppoli TA, Wulff AB, Cole AB, Thompson SM (2021) Harnessing psilocybin: antidepressant-like behavioral and synaptic actions of psilocybin are independent of 5-HT2R activation in mice. Proc Natl Acad Sci USA 118:e2022489118. 33850049 10.1073/pnas.2022489118 PMC8092378

[CIT0019] Heykants J , Van Peer A, Woestenborghs R, Gould S, Mills J (1986) Pharmacokinetics of ketanserin and its metabolite ketanserin-ol in man after intravenous, intramuscular and oral administration. Eur J Clin Pharmacol 31:343–350. 3792432 10.1007/BF00981135

[CIT0020] Holze F , Duthaler U, Vizeli P, Muller F, Borgwardt S, Liechti ME (2019) Pharmacokinetics and subjective effects of a novel oral LSD formulation in healthy subjects. Br J Clin Pharmacol 85:1474–1483. 30883864 10.1111/bcp.13918 PMC6595343

[CIT0021] Holze F , Vizeli P, Muller F, Ley L, Duerig R, Varghese N, Eckert A, Borgwardt S, Liechti ME (2020) Distinct acute effects of LSD, MDMA, and D-amphetamine in healthy subjects. Neuropsychopharmacology 45:462–471. 31733631 10.1038/s41386-019-0569-3 PMC6969135

[CIT0022] Holze F , Liechti ME, Hutten N, Mason NL, Dolder PC, Theunissen EL, Duthaler U, Feilding A, Ramaekers JG, Kuypers KPC (2021a) Pharmacokinetics and pharmacodynamics of lysergic acid diethylamide microdoses in healthy participants. Clin Pharmacol Ther 109:658–666. 32975835 10.1002/cpt.2057 PMC7984326

[CIT0023] Holze F , Vizeli P, Ley L, Muller F, Dolder P, Stocker M, Duthaler U, Varghese N, Eckert A, Borgwardt S, Liechti ME (2021b) Acute dose-dependent effects of lysergic acid diethylamide in a double-blind placebo-controlled study in healthy subjects. Neuropsychopharmacology 46:537–544. 33059356 10.1038/s41386-020-00883-6 PMC8027607

[CIT0024] Holze F , Ley L, Muller F, Becker AM, Straumann I, Vizeli P, Kuehne SS, Roder MA, Duthaler U, Kolaczynska K, Varghese N, Eckert A, Liechti ME (2022) Direct comparison of the acute effects of lysergic acid diethylamide and psilocybin in a double-blind placebo-controlled study in healthy subjects. Neuropsychopharmacology 47:1180–1187. doi:10.1038/s41386-022-01297-2. 35217796 PMC9018810

[CIT0025] Hutten N , Mason NL, Dolder P, Theunissen EL, Holze F, Liechti ME, Varghese N, Eckert A, Feilding A, Ramaekers JG, Kuypers KP (2020) Low doses of LSD acutely increase BDNF blood plasma levels in healthy volunteers. ACS Pharmacol Transl Sci 4:461–466. 33860175 10.1021/acsptsci.0c00099 PMC8033605

[CIT0026] Hysek CM , Liechti ME (2012) Effects of MDMA alone and after pretreatement with reboxetine, duloxetine, clonidine, carvedilol, and doxazosin on pupillary light reflex. Psychopharmacology (Berl) 224:363–376. 22700038 10.1007/s00213-012-2761-6

[CIT0027] Janke W , Debus G (1978) Die Eigenschaftswörterliste. Göttingen, Germany: Hogrefe.

[CIT0028] Koudas V , Nikolaou A, Hourdaki E, Giakoumaki SG, Roussos P, Bitsios P (2009) Comparison of ketanserin, buspirone and propranolol on arousal, pupil size and autonomic function in healthy volunteers. Psychopharmacology (Berl) 205:1–9. 19288084 10.1007/s00213-009-1508-5

[CIT0029] Krebs TS , Johansen PO (2012) Lysergic acid diethylamide (LSD) for alcoholism: meta-analysis of randomized controlled trials. J Psychopharmacol 26:994–1002. 22406913 10.1177/0269881112439253

[CIT0030] Liechti ME , Dolder PC, Schmid Y (2017) Alterations in consciousness and mystical-type experiences after acute LSD in humans. Psychopharmacology 234:1499–1510. 27714429 10.1007/s00213-016-4453-0 PMC5420386

[CIT0031] Ly C , Greb AC, Cameron LP, Wong JM, Barragan EV, Wilson PC, Burbach KF, Soltanzadeh Zarandi S, Sood A, Paddy MR, Duim WC, Dennis MY, McAllister AK, Ori-McKenney KM, Gray JA, Olson DE (2018) Psychedelics promote structural and functional neural plasticity. Cell Rep 23:3170–3182. 29898390 10.1016/j.celrep.2018.05.022 PMC6082376

[CIT0032] Madsen MK , Fisher PM, Burmester D, Dyssegaard A, Stenbaek DS, Kristiansen S, Johansen SS, Lehel S, Linnet K, Svarer C, Erritzoe D, Ozenne B, Knudsen GM (2019) Psychedelic effects of psilocybin correlate with serotonin 2A receptor occupancy and plasma psilocin levels. Neuropsychopharmacology 44:1328–1334. 30685771 10.1038/s41386-019-0324-9 PMC6785028

[CIT0033] Madsen MK , Knudsen GM, Knudsen GM (2021) Plasma psilocin critically determines behavioral and neurobiological effects of psilocybin. Neuropsychopharmacology 46:257–258. 32843702 10.1038/s41386-020-00823-4 PMC7688632

[CIT0034] Persson B , Heykants J, Hedner T (1991) Clinical pharmacokinetics of ketanserin. Clin Pharmacokinet 20:263–279. 2036747 10.2165/00003088-199120040-00002

[CIT0035] Preller KH , Herdener M, Pokorny T, Planzer A, Kraehenmann R, Stämpfli P, Liechti ME, Seifritz E, Vollenweider FX (2017) The fabric of meaning and subjective effects in LSD-induced states depend on serotonin 2A receptor activation. Curr Biol 27:451–457. 28132813 10.1016/j.cub.2016.12.030

[CIT0036] Rickli A , Moning OD, Hoener MC, Liechti ME (2016) Receptor interaction profiles of novel psychoactive tryptamines compared with classic hallucinogens. Eur Neuropsychopharmacol 26:1327–1337. 27216487 10.1016/j.euroneuro.2016.05.001

[CIT0037] Ross S , Bossis A, Guss J, Agin-Liebes G, Malone T, Cohen B, Mennenga SE, Belser A, Kalliontzi K, Babb J, Su Z, Corby P, Schmidt BL (2016) Rapid and sustained symptom reduction following psilocybin treatment for anxiety and depression in patients with life-threatening cancer: a randomized controlled trial. J Psychopharmacol 30:1165–1180. 27909164 10.1177/0269881116675512 PMC5367551

[CIT0038] Schmid Y , Enzler F, Gasser P, Grouzmann E, Preller KH, Vollenweider FX, Brenneisen R, Mueller F, Borgwardt S, Liechti ME (2015) Acute effects of lysergic acid diethylamide in healthy subjects. Biol Psychiatry 78:544–553. 25575620 10.1016/j.biopsych.2014.11.015

[CIT0039] Schmid Y , Gasser P, Oehen P, Liechti ME (2021) Acute subjective effects in LSD- and MDMA-assisted psychotherapy. J Psychopharmacol 35:362–374. 33853422 10.1177/0269881120959604

[CIT0040] Studerus E , Gamma A, Vollenweider FX (2010) Psychometric evaluation of the altered states of consciousness rating scale (OAV). PLoS One 5:e12412. 20824211 10.1371/journal.pone.0012412 PMC2930851

[CIT0041] Valle M , Maqueda AE, Rabella M, Rodriguez-Pujadas A, Antonijoan RM, Romero S, Alonso JF, Mananas MA, Barker S, Friedlander P, Feilding A, Riba J (2016) Inhibition of alpha oscillations through serotonin-2A receptor activation underlies the visual effects of ayahuasca in humans. Eur Neuropsychopharmacol 26:1161–1175. 27039035 10.1016/j.euroneuro.2016.03.012

[CIT0042] Vollenweider FX , Vollenweider-Scherpenhuyzen MF, Babler A, Vogel H, Hell D (1998) Psilocybin induces schizophrenia-like psychosis in humans via a serotonin-2 agonist action. Neuroreport 9:3897–3902. 9875725 10.1097/00001756-199812010-00024

[CIT0043] Wacker D , Wang S, McCorvy JD, Betz RM, Venkatakrishnan AJ, Levit A, Lansu K, Schools ZL, Che T, Nichols DE, Shoichet BK, Dror RO, Roth BL (2017) Crystal structure of an LSD-bound human serotonin receptor. Cell 168:377–389.e12.e312. 28129538 10.1016/j.cell.2016.12.033 PMC5289311

[CIT0044] Zerssen DV (1976) Die Beschwerden-Liste. Münchener Informationssystem. München, Germany: Psychis.

