# Supplementary material for: Ketanserin Reverses the Acute Response to LSD in a Randomized, Double-Blind, Placebo-Controlled, Crossover Study in Healthy Participants
Source: Int J Neuropsychopharmacol. 2022 Nov 7;26(2):97–106. doi: 10.1093/ijnp/pyac075 (PMC9926053; doi:10.1093/ijnp/pyac075)
Supplement: pyac075_suppl_Supplementary_Material [file pyac075_suppl_supplementary_material.pdf]

Supplement

Supplementary Methods

Ketanserin bioanalysis

Ketanserin was analysed by ultra-high performance liquid chromatography tandem mass spectrometry (UHPLC-MS/MS). The quaternary UHPLC system (Shimadzu, Kyoto, Japan) was connected to an API 5500 Qtrap tandem mass spectrometer (AB Sciex, Concord, Canada) equipped with a turbo ion spray source operated in the positive ionization mode. This is a validated method. Stability of the samples was ensured (samples were stored at -80°C and quickly extracted and analyzed).

Plasma sample aliquots of 50 µl were extracted with 150 µl acetonitrile containing 1 ng/ml risperidone, which was used as internal standard. Samples were mixed for 1 min and centrifuged for 30 min at 3220 g and 15° C. Samples were kept protected from light at 10°C in the autosampler and 2 µl supernatant was injected into the UHPLC-MS/MS system. The sample was pumped onto a Kinetex EVO C18 analytical column (1.7 µm 2.1x50 mm, Phenomenex, Torrance, USA) using 10% mobile phase B (acetonitrile 0.1% formic acid) and 90% mobile phase A (20 mM ammonium hydrogen carbonate in water adjusted to a pH of 9.0). A T-union was installed in front of the analytical column to mix the injected samples with mobile phase A, which was delivered by pump C. For the online dilution, the flow rate of pump A and B was increased from 0.1 ml/min to 0.6 ml/min and the flow rate of pump C was simultaneously decreased from 0.5 to 0 ml/min within the first 0.5 min of each run. The concentration of mobile phase B was linearly increased from 10% to 40% between 0.5 and 4.0 min. Afterwards, mobile phase B was increased to 95% in 0.5 min and kept at this concentration for another minute. Finally, the system was re-conditioned at 10% mobile phase B for another 0.5 min resulting in a total run time of 6 min. Chromatographic separation was performed at 30°C resulting in a retention time of 3.7 and 4.2 min for risperidone and ketanserin, respectively. Analytes were detected by multiple reaction monitoring using as quantifier mass transition m/z 396.1→189.1 for ketanserin and 411.1→191.1 for risperidone. The transition m/z 396.1→146.1 and m/z 411.1→110.0 were used as qualifiers for ketanserin and risperidone, respectively. Analyte specific settings are depict in Supplementary Table S5. Nitrogen was used as collision gas (medium), curtain gas (20 psi), ion source gas 1 (30 psi) and ion source gas 2 (60 psi). The source temperature was set to 500°C and the ion spray voltage to 5500 V. Calibration and quality control (QC) samples were prepared in human plasma. The calibration range of ketanserin was 0.25-500 ng/ml. Four QC levels (0.25, 2.5, 25, 250 ng/ml) with 4 to 6 replicates were included for each analytical run. The intra-assay accuracy and precision of QC samples had to be between 85-115% (lower limit of quantification [LLOQ]: 80-120%) and ≤15% (LLOQ: ≤20%), respectively. At least 10% of all samples were re-analysed, which resulted in a bias between original and reanalysis of ≤10%.

Sample size calculation

Power analysis was performed with PASS® (Hintze J. Kaysville, UT, USA). For the primary outcome, which was the duration of the LSD effect, we expected that ketanserin would decrease the LSD response duration by 3 h from 8.5 to 5.5 h. For the effect duration endpoint, a sample size of 10 achieved 80% power to detect a difference of 3.0 between the null hypothesis mean of 8.5 and the alternative hypothesis mean of 5.5 with an estimated standard deviation of 3.0 (Holze et al., 2019) and with a significance level (alpha) of 0.05 using a two-sided one-sample *t*-test. For the co-primary endpoints, we expected decreases in area under the effect curve (AUEC) and E_{max} values of the LSD effect by 30 and 15%, respectively. For the AUEC, a sample size of 10 achieved 80% power to detect a difference of 30% between the null hypothesis mean of 100% and the alternative hypothesis mean of 70% with an

estimated standard deviation of 30% and with a significance level (alpha) of 0.05 using a two-sided one-sample *t*-test. For the E_{max}, a sample size of 16 achieved 80% power to detect a difference of 15% between the null hypothesis mean of 100% and the alternative hypothesis mean of 85% with an estimated standard deviation of 20% and with a significance level (alpha) of 0.05 using a two-sided one-sample *t*-test.

Subjective drug effects measurements

Visual Analog Scales (VASs)

Subjective effects were assessed repeatedly using visual analog scales (VASs) (Schmid et al., 2015; Holze et al., 2020; Holze et al., 2021b; Holze et al., 2022) 0, 0.5, 1, 1.5, 2, 2.5, 3, 3.5, 4, 5, 6, 7, 8, 9, 10, 11, and 12 h after LSD administration (VASs were assessed each time LSD blood concentrations were measured). The VASs included “any drug effect,” “good drug effect,” “bad drug effect,” “stimulated”, “fear”, “ego-dissolution”, “nausea”, “tiredness”, “visual alterations”, “auditory alterations”, “synesthesia”, “alteration in time perception”, and “insight,” that were presented as 100-mm horizontal lines (0-100 %), marked from “not at all” (0 mm) on the left to “extremely” (100 mm) on the right (Hysek et al., 2014; Holze et al., 2020). Further VASs included “talkative,” “trust,” “open”, and “inward focus” which were bidirectional and marked with “normal” in the middle at 0 mm and “not at all” (-50 mm) on the left and “extremely” (50 mm) on the right. The primary VAS outcome measure was “any drug effect” which was also used to calculate the characteristics of the LSD response over time as done previously (Holze et al., 2019; Holze et al., 2020; Holze et al., 2021a; Holze et al., 2021b; Holze et al., 2022). The VAS can relatively rapidly and easily be completed by the participant even during the LSD experience and allowing for a valid prospective definition of the drug effects over time. They are sensitive and relatively simple measures. More complex assessments of the LSD state have to be performed primarily at the end of the session and include entire multi-item questionnaires. The VAS “any drug effect” is an overall effect measure to characterize the overall effect intensity and time course. The VAS “good drug effect” is an overall measure of effects subjectively considered positive and interrelated with other measures such as “drug liking”. The VAS “bad drug effect” is an overall measure of any negative effects and related to “fear”. Typically, “bad drug effects” tend to occur only at higher doses or plasma concentrations according to previous PK-PD analyses (Dolder et al., 2017; Holze et al., 2019). The VAS “ego dissolution” was marked with the sentence: “the boundaries between myself and my surroundings seemed to blur”. This is also an item of the 5D-ASC (no. 71) which has been used as a simple measure of “ego dissolution” previously (Tagliazucchi et al., 2016; Liechti et al., 2017) and can be used repeatedly as a single VAS (Holze et al., 2019; Holze et al., 2020).

Adjective Mood Rating Scale (AMRS)

The Adjective Mood Rating Scale (AMRS) (Janke and Debus, 1978) was used 1 h before and 3, 6, 9, 12, and 24 h after drug administration. The AMRS is a validated 60-item Likert mood rating scale mainly used in Europe and consists of subscales including ratings on “concentration”, inactivity”, “extraversion”, “introversion”, “well-being”, “emotional excitation”, “anxiety”, and “dreaminess”. It is suitable for repeated measurements of mood states. The short German EWL60S version was used (Janke and Debus, 1978). The completion of the ratings under the effects of psychedelics substances is possible but difficult because it lasts several minutes. The scale was used in paper and pencil by the participants and, if completion was not possible, verbally by the investigator during states of markedly impaired concentration. The AMRS was included as a secondary supportive measure to the findings on the VAS because it was considered a better validated measure of mood states and produced more defined ratings than the VAS (AMRS well-being considered similar to VAS good drug effects; AMRS anxiety considered similar to VAS fear).

5 Dimensions of Altered States of Consciousness (5D-ASC) scale

The 5 Dimensions of Altered States of Consciousness (5D-ASC) scale (Dittrich, 1998; Studerus et al., 2010) was used as secondary outcome measure and as primary measure of the psychedelic-typical peak alterations of the mind and was administered 12 h after LSD to retrospectively rate the overall psychedelic experience at its peak. The 5D-ASC scale measures altered states of consciousness and contains 94 items (visual analog scales). The instrument consists of five subscales/dimensions (Dittrich, 1998) and 11 lower-order scales (Studerus et al., 2010). The 5D-ASC dimension “Oceanic Boundlessness” (27 items) measures derealization and depersonalization associated with positive emotional states, ranging from heightened mood to euphoric exaltation. The corresponding lower-order scales include “experience of unity”, “spiritual experience”, “blissful state”, “insightfulness”, and “disembodiment”. The dimension “Anxious Ego Dissolution” (21 items) summarizes ego-disintegration and loss of self-control phenomena associated with anxiety. The corresponding lower-order scales include “impaired control of cognition” and “anxiety.” The dimension “Visionary Restructuralization” (18 items) consists of the lower-order scales “complex imagery,” “elementary imagery,” “audio-visual synesthesia,” and “changed meaning of percepts.” Two additional dimensions describe “Auditory Alterations” (15 items) and “Reduction of Vigilance” (12 items). The total 3D-ASC score is the total of the three main dimensions “Oceanic Boundlessness”, “Anxious Ego-Dissolution”, and “Visionary Restructuralization” and can be used as a measure of the overall intensity of the alteration of the mind (Liechti et al., 2017). The scale is well-validated in German (Dittrich, 1998) and many other languages and widely used to characterize the subjective effects of various psychedelic drugs. In particular, the scale has been used by most research groups to psychometrically assess LSD effects (Schmid et al., 2015; Carhart-Harris et al., 2016; Dolder et al., 2016; Preller et al., 2017; Bershad et al., 2019; Holze et al., 2019; Holze et al., 2020; Holze et al., 2021b; Holze et al., 2022). Furthermore, acute ratings on the 5D-ASC after administration of psilocybin have been used to predict long-term effects of psychedelic treatments in patients (Griffiths et al., 2016; Roseman et al., 2017). Ratings on the 5D-ASC have been shown to closely correlate with ratings on the Mystical Effects Questionnaire (MEQ, see below) (Liechti et al., 2017) which is primarily used by research groups in the US (Griffiths et al., 2016).

Mystical Effects Questionnaire (MEQ30)

Mystical experiences were assessed 12 h after LSD administration using the 100-item States of Consciousness Questionnaire (SOCQ) (Griffiths et al., 2006; Liechti et al., 2017) that includes the 30-item Mystical Effects Questionnaire (MEQ30) (Barrett et al., 2015), and subscales for “aesthetic experience” and negative “nadir” effects. The published German version was used (Liechti et al., 2017). The MEQ has been used in numerous experimental and therapeutic trials with psilocybin (Griffiths et al., 2006; Griffiths et al., 2008; Griffiths et al., 2011; MacLean et al., 2011; Garcia-Romeu et al., 2014; Griffiths et al., 2016; Ross et al., 2016; Griffiths et al., 2018; Garcia-Romeu et al., 2019). We derived the four scale scores: mystical experience, positive mood, transcendence of time and space, and ineffability (Barrett et al., 2015). The total of all scale scores was used as an overall measure of the mystical-type experience. A complete mystical experience was defined as scores ≥ 60% on all MEQ30 factors (Barrett et al., 2015). While we prefer the German 5D-ASC scale, the German version of the MEQ was also included to facilitate comparison of our findings with those from research using the MEQ (mainly US). Additionally, some aspects of the LSD experience may be better captures with this scale. For the scale validation see (Barrett et al., 2015). For an analysis of the interrelation of the two measures with regards to responses to LSD see (Liechti et al., 2017). For the German translation of the MEQ30 see online supplement of (Liechti et al., 2017).

Autonomic effects

Blood pressure, heart rate and body temperature were assessed repeatedly 0, 0.5, 1, 1.5, 2, 2.5, 3, 3.5, 4, 5, 6, 7, 8, 9, 10, 11, and 12 h after LSD administration. Blood pressure

(systolic and diastolic) and heart rate were measured using an automatic oscillometric device (OMRON Healthcare Europe NA, Hoofddorp, Netherlands). The measurements were performed in duplicate at an interval of 1 min and after a resting time of at least 10 min. The averages were calculated for analysis. Core (tympanic) temperature was measured using an GENIUSTM 2 ear thermometer (Tyco Healthcare Group LP, Watertown, NY, USA).

Pupillometry was performed before and 1, 2.5, 4, 7, and 11 h after LSD administration using an infrared pupillometer (PRL-200, NeurOptics, Irvine, CA, USA) under standardized dark-light conditions as previously described (Hysek and Liechti, 2012). The dark-adapted maximal pupil diameter, minimal pupil diameter after a light stimulus, and constriction amplitude (difference between maximal and minimal pupil size) were recorded.

Supplementary Results

Participants

Prior substance use

Eleven participants have had experiences with hallucinogenic substances, three of which had used LSD before (1-2 times). Thirteen had used 3,4-methylenedioxymethamphetamine (MDMA) (1-30 times). Twelve participants had used stimulants before including cocaine (7 participants, 1-30 times), amphetamine (5 participants, 1-10 times), and methylphenidate (5 participants, 1-20 times). Seven participants had used nitrous oxide before (1-5 times), two of which had used ketamine as well (1-2 times).

Supplementary Tables

Table S1. Mean values and statistics for the acute effects of LSD on the Adjective Mood Rating Scale (AMRS)

		Ketanserin mean ± SEM	Placebo mean ± SEM	t_{23}	p
Concentration	ΔE_{max}	-0.4 ± 0.4	-1.2 ± 0.5	1.5	0.157
	AUEC	126 ± 3	117 ± 5	2.1	**0.043 ***
General Inactivity	ΔE_{max}	9.2 ± 1.6	12.4 ± 1.8	- 1.7	0.110
	AUEC	485 ± 14	518 ± 18	- 1.8	0.080
Extraverion	ΔE_{max}	0.5 ± 0.4	-0.6 ± 0.5	2.0	0.063
	AUEC	131 ± 4	119 ± 5	2.1	**0.047 ***
Introversion	ΔE_{max}	2.9 ± 0.7	5.0 ± 0.6	- 2.8	**0.010** *
	AUEC	155 ± 4	175 ± 6	- 3.6	**0.001** **
Well-being	ΔE_{max}	1.4 ± 0.8	2.2 ± 0.7	- 1.1	0.273
	AUEC	275 ± 8	272 ± 9	0.4	0.704
Emotional excitation	ΔE_{max}	0.0 ± 1.0	3.7 ± 1.0	- 3.9	**0.001 ****
	AUEC	413 ± 5	440 ± 8	- 3.6	**0.001 ****
Anxiety	ΔE_{max}	0.3 ± 0.5	0.7 ± 0.3	- 1.0	0.342
	AUEC	141 ± 2	146 ± 2	- 1.8	0.093
Dreaminess	ΔE_{max}	3.8 ± 0.6	6.5 ± 0.6	- 4.1	**0.000 *****
	AUEC	160 ± 4	186 ± 5	- 5.7	**0.000 *****

*p<0.05, **p<0.01, ***p<0.001; AUEC, Area under the effect curve; SEM, standard error of the mean; ΔE_{max}, maximal difference from baseline; N=24.

Table S2. Acute alterations of mind induced by LSD on retrospective assessment scales

		Ketanserin<br>mean ± SEM	Placebo<br>mean ± SEM	t_{23}	p
5 Dimensions of Altered States of Consciousness (ASC) Scale					
Oceanic boundlessness	% score	40 ± 4	60 ± 5	- 4.1	**0.000** ***
Anxious ego-dissolution	% score	16 ± 3	22 ± 4	- 1.9	0.077
Visionary restructuralization	% score	47 ± 5	60 ± 5	- 2.7	**0.014** *
Auditory alterations	% score	14 ± 3	22 ± 5	- 1.9	0.073
Reductions of vigilance	% score	40 ± 5	51 ± 6	- 2.4	**0.026** *
5D-ASC total score	% score	32 ± 3	45 ± 4	- 3.9	**0.001** **
3D-OAV total score	% score	34 ± 3	48 ± 4	- 3.9	**0.001** **
Experience of unity	% score	44 ± 6	66 ± 6	- 3.3	**0.003** **
Spiritual experience	% score	17 ± 4	37 ± 6	- 2.9	**0.008** **
Blissful state	% score	54 ± 6	67 ± 6	- 2.0	0.058
Insightfulness	% score	25 ± 5	49 ± 6	- 5.2	**0.000** ***
Disembodiment	% score	31 ± 6	50 ± 6	- 2.4	0.026
Impaired control and cognition	% score	29 ± 4	38 ± 5	- 1.7	0.105
Anxiety	% score	6 ± 4	11 ± 4	- 1.7	0.098
Complex imagery	% score	50 ± 7	67 ± 6	- 1.9	0.064
Elementary imagery	% score	55 ± 6	69 ± 7	- 1.6	0.122
Audio-visual synesthesia	% score	65 ± 8	75 ± 6	- 1.2	0.254
Changed meaning of percepts	% score	42 ± 6	46 ± 7	- 0.5	0.600
Mystical Effects Questionnaire (MEQ30)					
Mystical	% score	39 ± 6	37 ± 4	0.4	0.683
Positive mood	% score	57 ± 5	54 ± 4	0.5	0.615
Transcendence of time/space	% score	51 ± 6	63 ± 5	- 1.9	0.070
Ineffability	% score	61 ± 6	73 ± 5	- 1.6	0.125
MEQ30 total score	% score	47 ± 5	49 ± 3	- 0.3	0.736
Nadir	% score	17 ± 3	16 ± 3	0.2	0.849
Aesthetic Experience	% score	35 ± 4	55 ± 5	- 3.2	**0.004** **

*p<0.05, **p<0.01, ***p<0.001; SEM, standard error of the mean; N=24.

Table S3. Mean values and statistics for acute autonomic and adverse effects of LSD

		Ketanserin mean ± SEM	Placebo mean ± SEM	t_{23}	p
Vital signs					
Heart rate (beats/min)	E_{max}	82 ± 2	81 ± 21	0.5	0.594
	AUEC	794 ± 18	815 ± 18	- 2.1	0.052
Systolic blood pressure (mmHg)	E_{max}	136 ± 3	137 ± 2	- 0.4	0.660
	AUEC	1417 ± 26	1491 ± 25	- 3.9	**0.001** **
Diastolic blood pressure (mmHg)	E_{max}	84 ± 1	88 ± 1	- 3.0	**0.006** **
	AUEC	847 ± 11	922 ± 12	- 7.2	**0.000** ***
Rate pressure product	E_{max}	10.6 ± 0.3	10.7 ± 0.3	- 0.2	0.851
	AUEC	96 ± 6	103 ± 2	- 3.7	**0.001** **
Mean arterlial pressure	E_{max}	98 ± 1	99 ± 1	- 0.6	0.557
	AUEC	1020 ± 12	1076 ± 12	- 5.3	**0.000** ***
Body temperature (°C)	E_{max}	37.5 ± 0.1	37.5 ± 0.1	- 0.6	0.582
	AUEC	435 ± 1	436 ± 1	- 0.9	0.359
Pupil dialation (mm)					
Maximum diameter	E_{max}	6.6 ± 0.2	6.8 ± 0.2	- 3.1	**0.005** **
	AUEC	64 ± 2	72 ± 2	-10.2	**0.000** ***
Minimum diameter	E_{max}	5.4 ± 0.2	6.1 ± 0.2	- 6.5	**0.001** **
	E_{min}	3.7 ± 0.2	4.5 ± 0.2	- 8.3	**0.000** ***
	AUEC	46 ± 2	54 ± 2	-11.2	**0.000** ***
Constriction	E_{max}	1.8 ± 0.1	1.7 ± 0.1	4.0	**0.001** **
	E_{min}	1.4 ± 0.1	1.4 ± 0.1	1.0	0.319
	AUEC	18 ± 1	17 ± 1	3.1	**0.005** **
List of Complaints (LC score)					
Compaints at baseline	-1h	2 ± 0	1 ± 0	0.9	0.388
Acute adverse effects	$\Delta E_{0\text{-}12h}$	10 ± 2	13 ± 2	1.5	0.135

*p<0.05, **p<0.01, ***p<0.001; AUEC, Area under the effect curve; E_{max}, maximal effect; SEM, standard error of the mean; $\Delta E_{0\text{-}12h}$, difference from baseline; N=24.

Table S4. Mean values and statistics for Brain-Derived Neurotrophic Factor (BDNF) plasma concentrations (pg/mL)

	Ketanserin mean ± SEM	Placebo mean ± SEM	t_{23}	p
0 h	3.1 ± 1.0	3.7 ± 1.3	- 0.9	0.395
6 h	3.4 ± 1.0	3.7 ± 1.2	- 0.4	0.664
9 h	3.2 ± 1.1	3.9 ± 1.2	- 1.3	0.211
12 h	4.2 ± 1.0	3.6 ± 1.1	1.1	0.285
C_{max}	5.0 ± 1.1	4.8 ± 1.2	0.3	0.803
ΔC_{max}	1.9 ± 0.4	1.2 ± 0.3	1.4	0.188

C_{max}, maximal concentration ΔC_{max}, maximal difference from baseline (0 h); N=24.

Table S5. Mass spectrometry settings of ketanserin and risperidone

	Q1 mass (Da)	Q3 mass (Da)	Dwell time (msec)	DP (V)	EP (V)	CE (V)	CXP (V)
Ketanserin I	396.082	189.1	50	111	10	20	12
Ketanserin II	396.082	146.1	50	111	10	32	10
Risperidone I	411.101	191.1	50	146	10	39	12
Risperidone II	411.101	110.0	50	146	10	67	14

CE, Collision energy; CXP, Collision cell exit potential; DP, Declustering potential; EP, Entrance potential; N=24.

Supplementary Figures

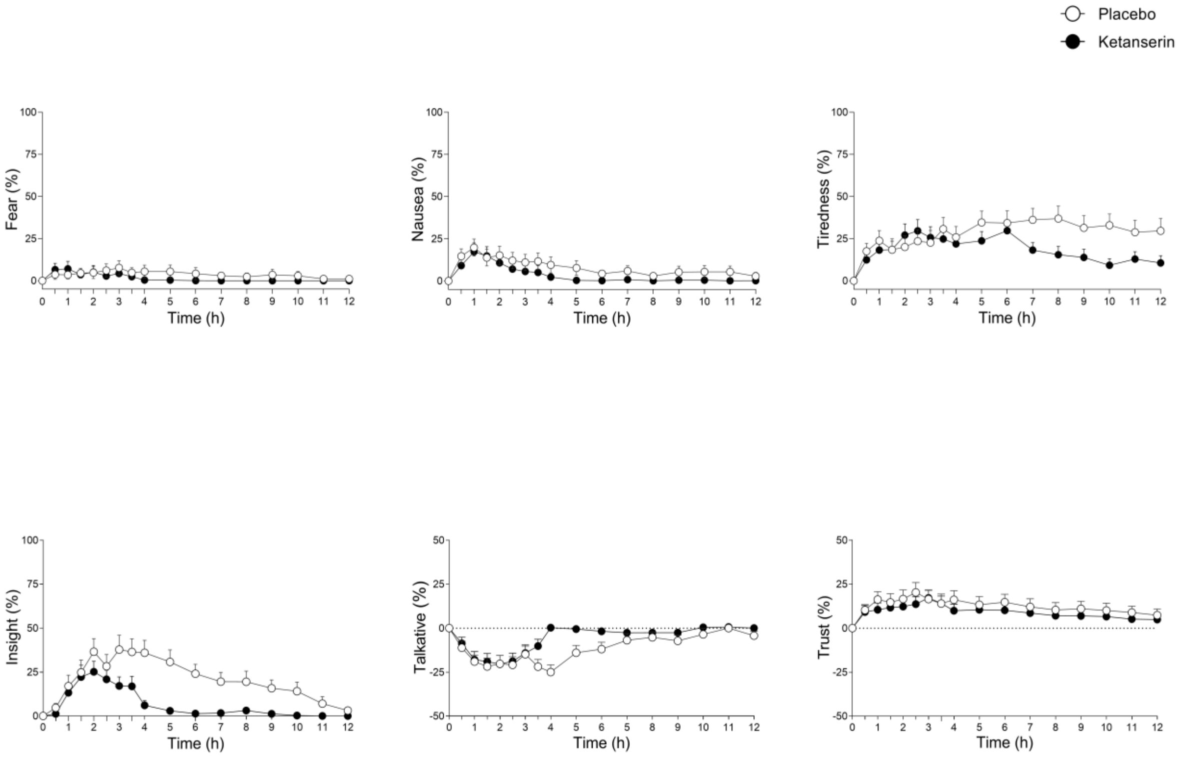

Figure S1. Subjective effects of LSD over time on Visual Analog Scales (VASs). Ketanserin significantly reduced LSD-induced subjective effects compared with placebo as evidenced by reduced area under the effect curve (AUEC) values for “nausea,” “tiredness,” “insight,” and “talkative”. LSD was administered at t = 0 h. Ketanserin or placebo was administered at t = 1 h. LSD did not relevantly increase “fear” and therefore ketanserin had no effects. LSD slightly elevated feelings of trust, which was not significantly reduced by ketanserin. The data are expressed as the mean ± SEM in 24 subjects. Additional subjective effects are shown in Figure 1. The corresponding maximal responses and AUEC values and statistics are shown in Table 2.

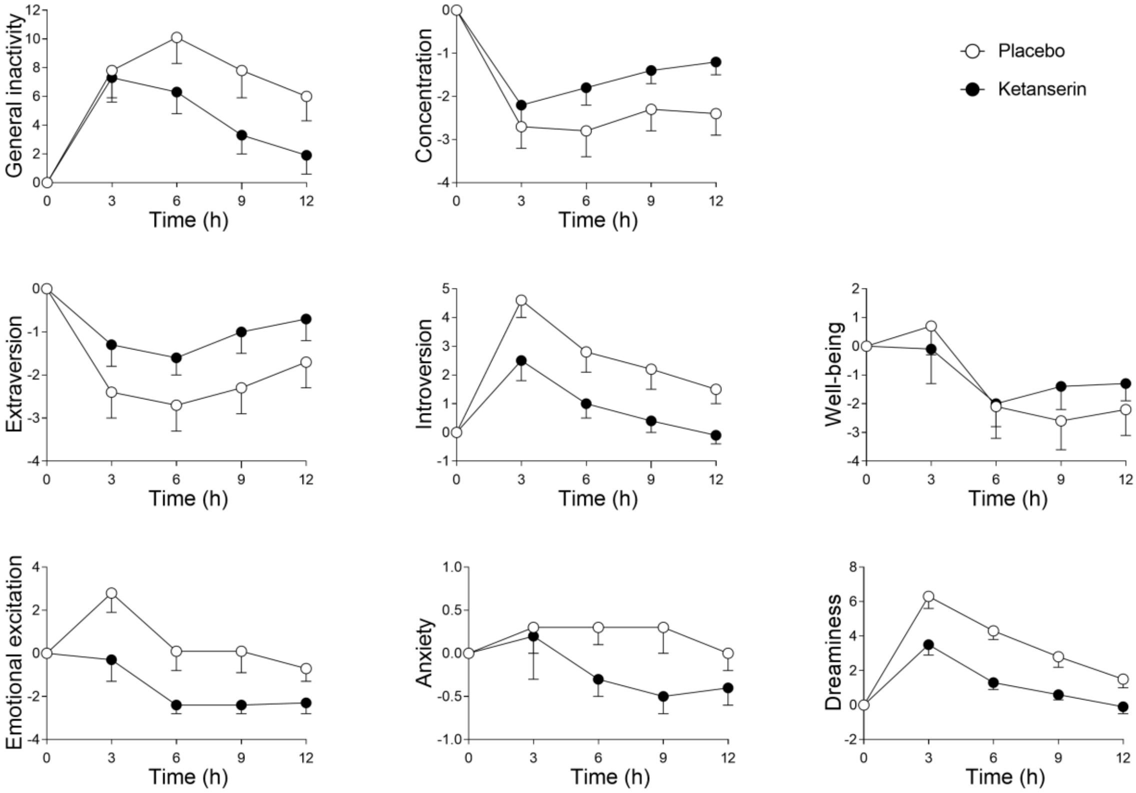

Figure S2. Subjective effects of LSD over time on the Adjective Mood Rating Scale (AMRS). Ketanserin significantly reduced LSD-induced elevations in mood ratings compared with placebo as evidenced by reduced area under the effect curve (AUEC) values for “concentration,” “introversion,” “emotional excitation,” and “dreaminess,” and increased the rating for “extraversion”. LSD increased “inactivity” compared with baseline which was not significantly decreased by ketanserin. Anxiety ratings were not affected by ketanserin which is likely due to the minimal increase by LSD compared with baseline. Ketanserin had no effect on well-being compared with placebo. LSD was administered at t = 0 h. Ketanserin or placebo was administered at t = 1 h. The data are expressed as the mean ± SEM in 24 subjects. The corresponding maximal responses and statistics are shown in Supplementary Table S1.

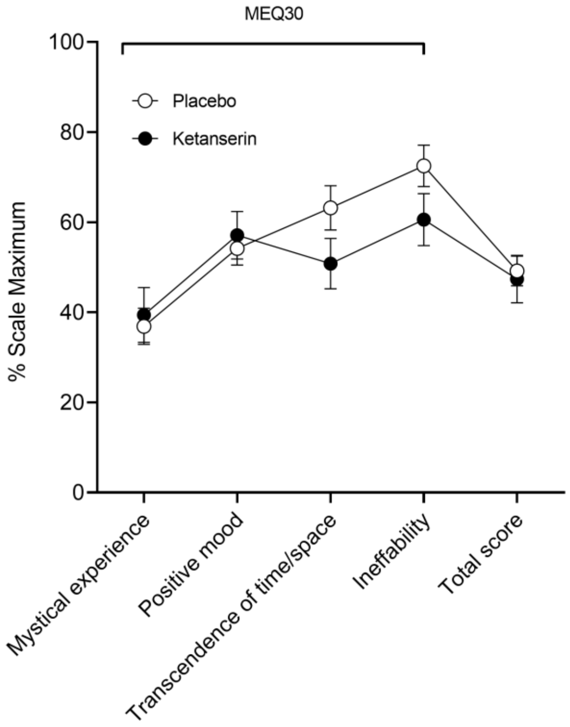

Figure S3. Effects of LSD on the Mystical Experiences Questionnaire (MEQ) 30. LSD increased ratings on the MEQ30 scale. Ketanserin had no effect on the mystical experience associated with LSD, as assessed with the MEQ 30 total score. The data are expressed as the mean ± SEM percentage of maximally possible scale scores in 24 subjects. The corresponding statistics are shown in Supplementary Table S2.

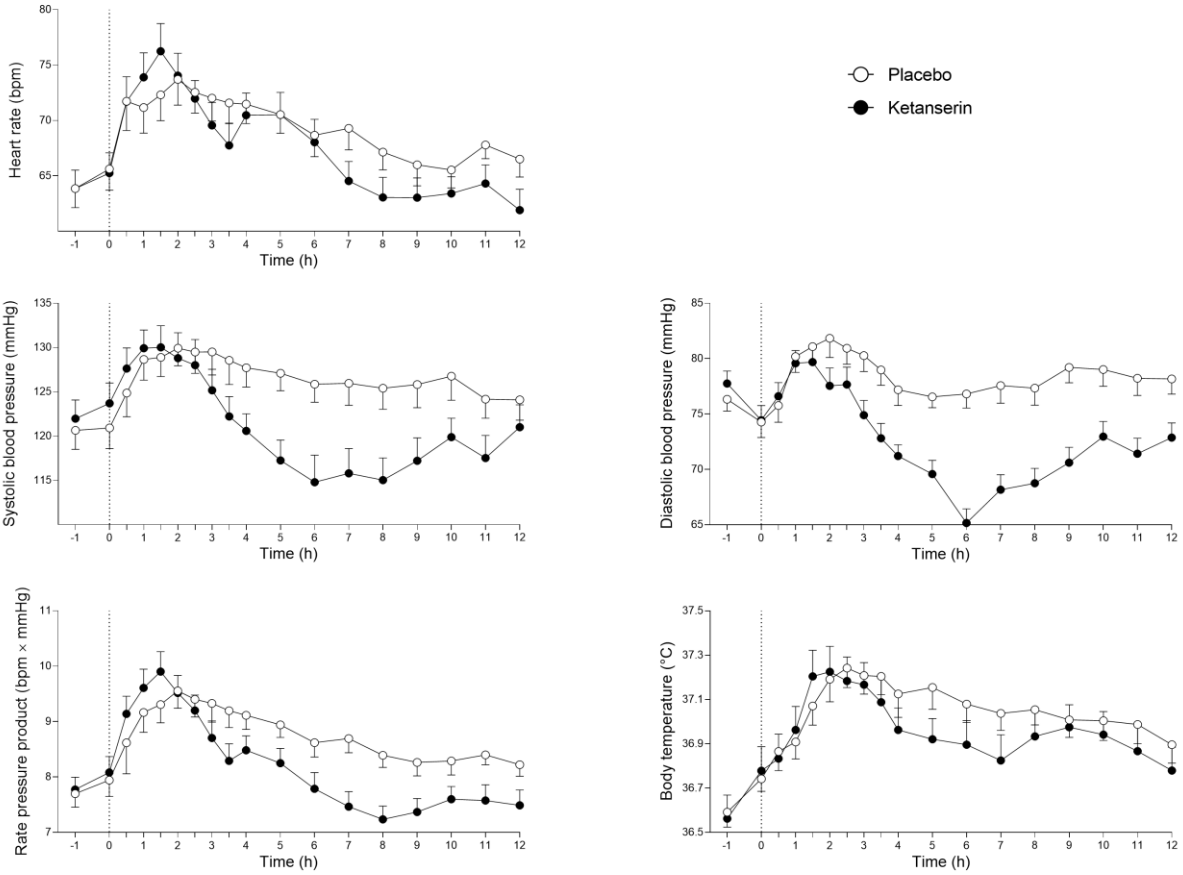

Figure S4. Autonomic effects of LSD. LSD increased heart rate, blood pressure, rate pressure product, and body temperature over time and compared with baseline (t = 0 h). Ketanserin did not significantly reduce overall heart rate compared with placebo (AUEC reduction). However, ketanserin significantly reduced LSD-induced elevations in systolic blood pressure and diastolic blood pressure. Ketanserin also significantly reduced the rate pressure product. Ketanserin had no significant effect on the body temperature elevation associated with LSD. LSD was administered at t = 0 h. Ketanserin or placebo was administered at t = 1 h. The data are expressed as the mean ± SEM in 24 subjects. The corresponding statistics are shown in Supplementary Table S3.

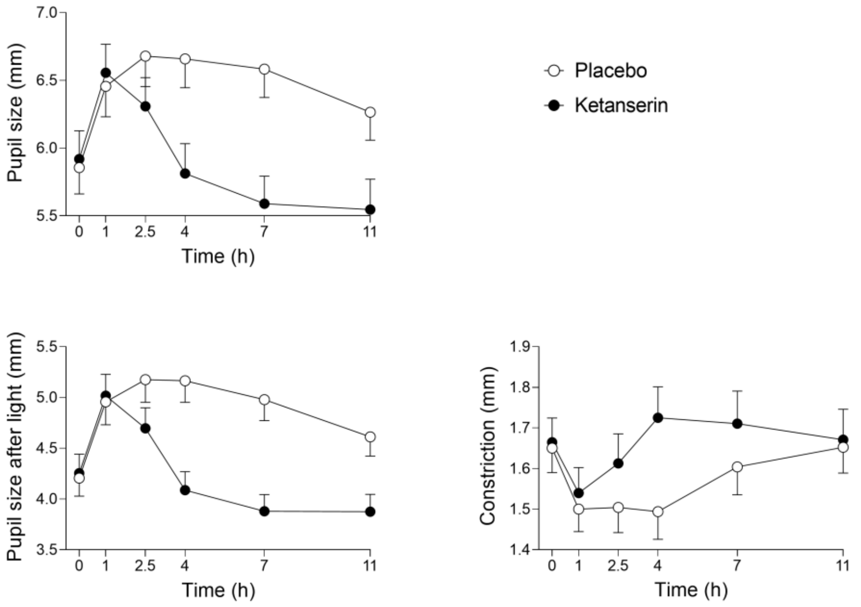

Figure S5. Effects of LSD on pupillary function. LSD increased pupil size at rest and after a light stimulus and the constriction amplitude compared to baseline (t = 0 h). Ketanserin significantly reversed the LSD-induced effects on pupil size at rest and after a light stimulus as well as the effect of LSD on the constriction. LSD was administered at t = 0 h. Ketanserin or placebo was administered at t = 1 h. The data are expressed as the mean ± SEM in 24 subjects. The corresponding statistics are shown in Supplementary Table S3.

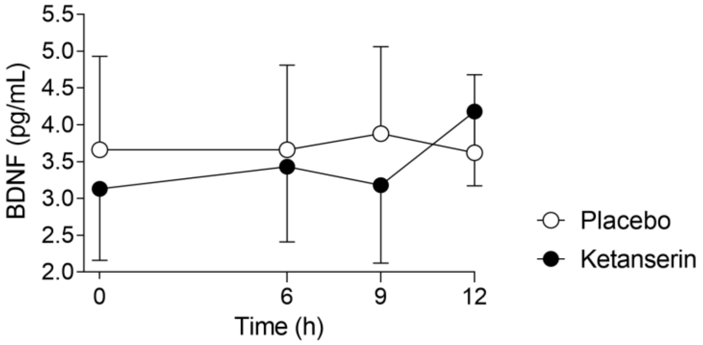

Figure S6. LSD effects on circulating brain-derived neurotrophic factor (BDNF). LSD moderately increased peak plasma concentrations of BDNF compared with baseline (t = 0 h). Ketanserin did not alter the BDNF increase associated with LSD compared with placebo. LSD was administered at t = 0 h. Ketanserin or placebo was administered at t = 1 h. The data are expressed as the mean ± SEM in 24 subjects. The corresponding statistics are shown in Supplementary Table S4.

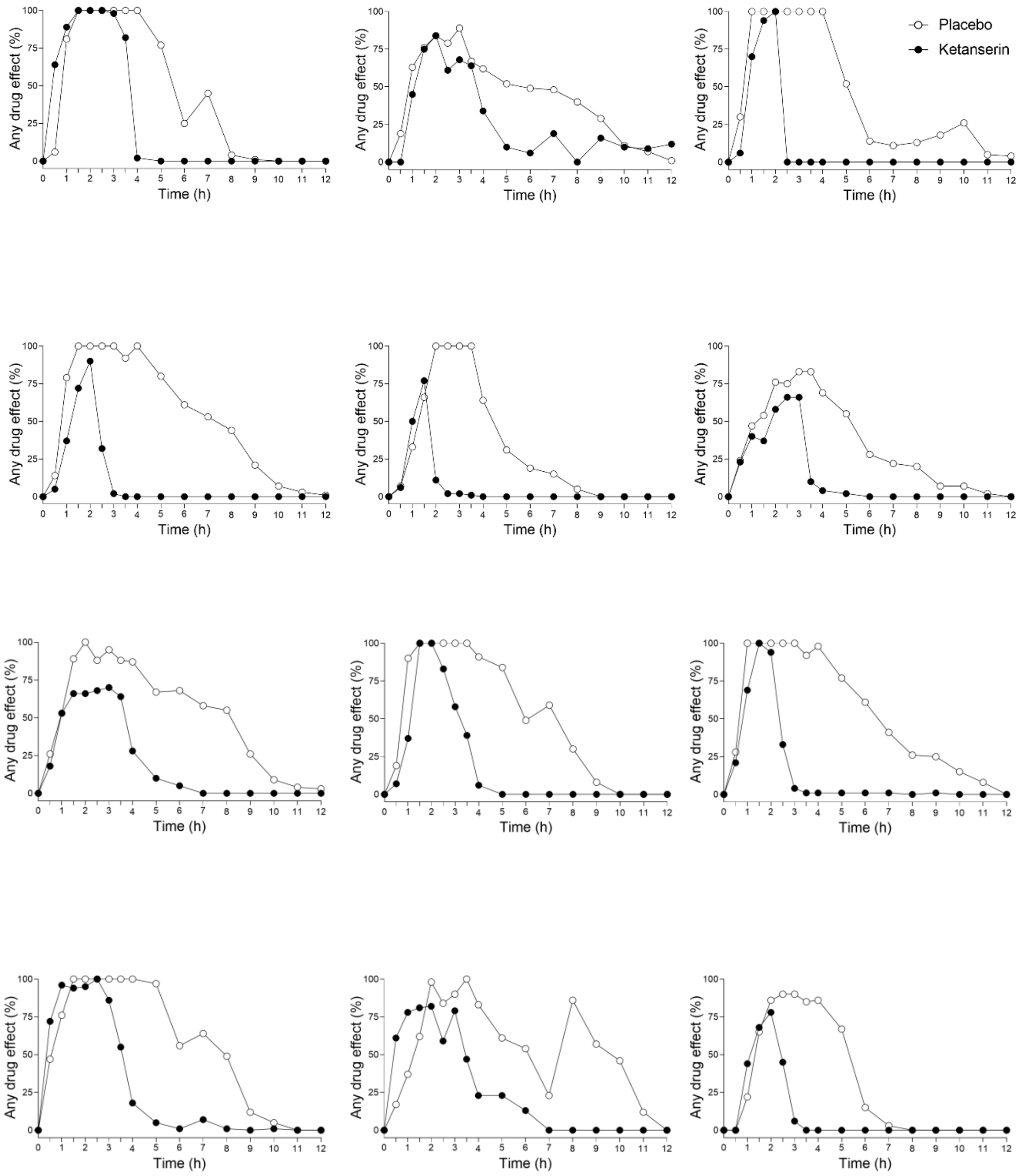
Placebo
Ketanserin
Any drug effect (%)
Time (h)

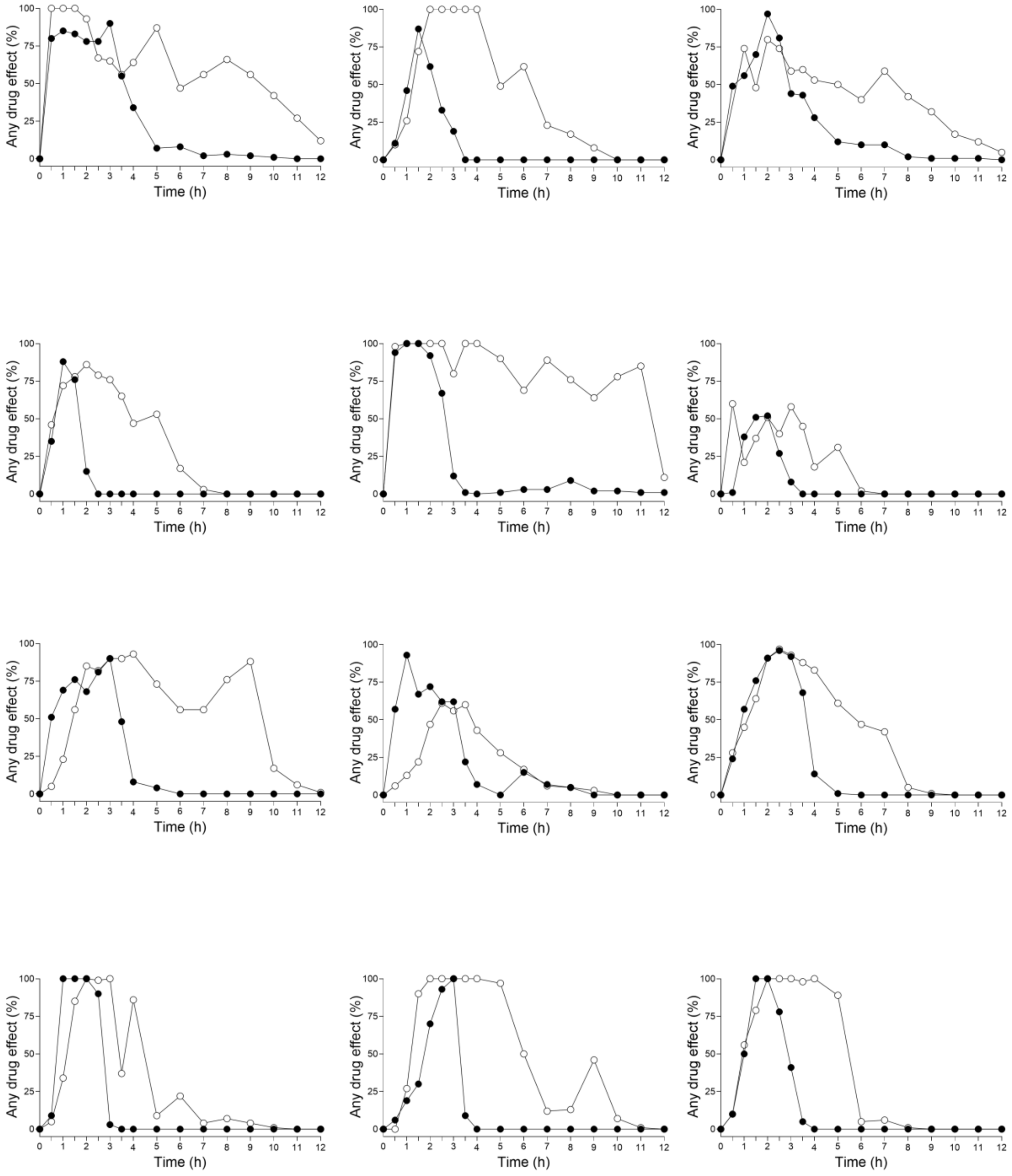

Figure S7. Individual effect-time curves of subjective "any drug effect" of LSD. Ketanserin significantly reduced the duration of the Visual Analog Scale (VAS) "any drug effect" which was the primary predefined outcome measure. The data are expressed as single ratings at each time-point in 24 subjects (12 men, 12 women). LSD was administered at t = 0 h. Ketanserin or placebo was administered at t = 1 h. Mean subjective effects are shown in Figure 1. The corresponding maximal effect and AUEC values and statistics are shown in Table 1.

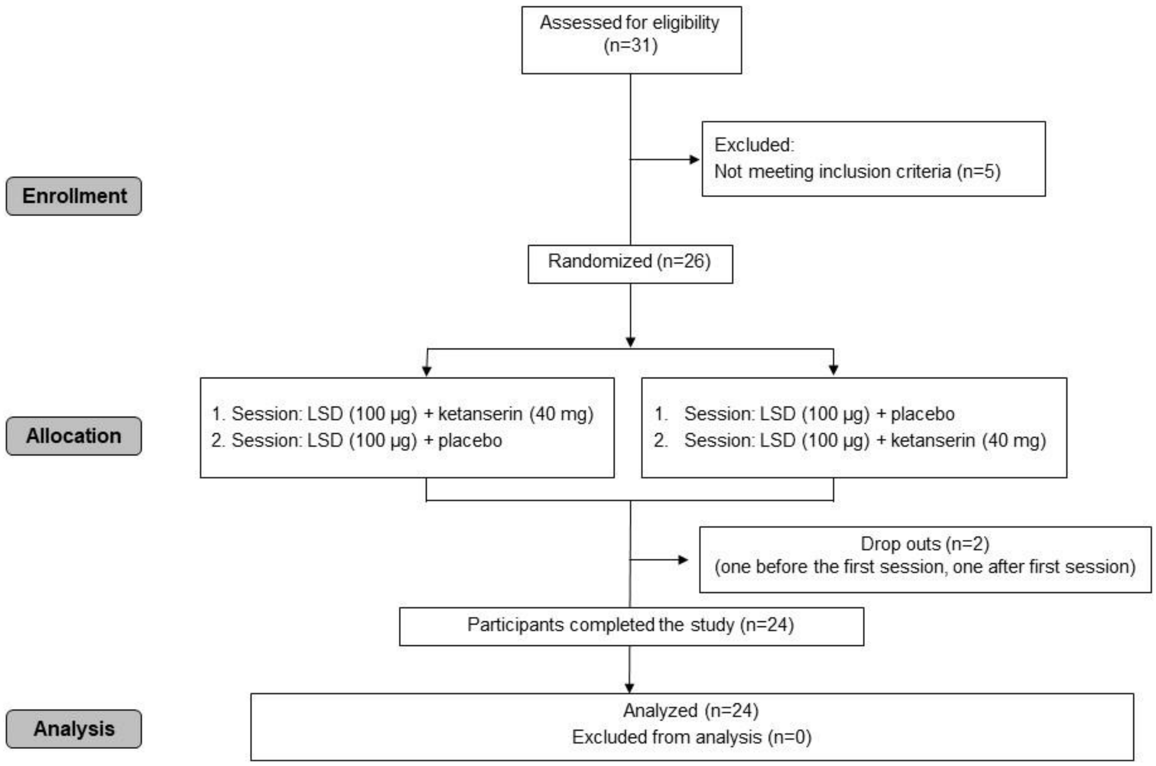

Figure S8. "CONSORT" Flow Chart. Progress of all participants in the study.

References

Barrett FS, Johnson MW, Griffiths RR (2015) Validation of the revised Mystical Experience Questionnaire in experimental sessions with psilocybin. J Psychopharmacol 29:1182-1190.

Bershad AK, Schepers ST, Bremmer MP, Lee R, de Wit H (2019) Acute subjective and behavioral effects of microdoses of lysergic acid diethylamide in healthy human volunteers. Biol Psychiatry 86:792-800.

Carhart-Harris RL, Kaelen M, Bolstridge M, Williams TM, Williams LT, Underwood R, Feilding A, Nutt DJ (2016) The paradoxical psychological effects of lysergic acid diethylamide (LSD). Psychol Med 46:1379-1390.

Dittrich A (1998) The standardized psychometric assessment of altered states of consciousness (ASCs) in humans. Pharmacopsychiatry 31 (Suppl 2):80-84.

Dolder PC, Schmid Y, Mueller F, Borgwardt S, Liechti ME (2016) LSD acutely impairs fear recognition and enhances emotional empathy and sociality. Neuropsychopharmacology 41:2638-2646.

Dolder PC, Schmid Y, Steuer AE, Kraemer T, Rentsch KM, Hammann F, Liechti ME (2017) Pharmacokinetics and pharmacodynamics of lysergic acid diethylamide in healthy subjects. Clin Pharmacokinetics 56:1219-1230.

Garcia-Romeu A, Griffiths RR, Johnson MW (2014) Psilocybin-occasioned mystical experiences in the treatment of tobacco addiction. Curr Drug Abuse Rev 7:157-164.

Garcia-Romeu A, Davis AK, Erowid F, Erowid E, Griffiths RR, Johnson MW (2019) Cessation and reduction in alcohol consumption and misuse after psychedelic use. J Psychopharmacol 33:1088-1101.

Griffiths R, Richards W, Johnson M, McCann U, Jesse R (2008) Mystical-type experiences occasioned by psilocybin mediate the attribution of personal meaning and spiritual significance 14 months later. J Psychopharmacol 22:621-632.

Griffiths RR, Richards WA, McCann U, Jesse R (2006) Psilocybin can occasion mystical-type experiences having substantial and sustained personal meaning and spiritual significance. Psychopharmacology (Berl) 187:268-283.

Griffiths RR, Johnson MW, Richards WA, Richards BD, McCann U, Jesse R (2011) Psilocybin occasioned mystical-type experiences: immediate and persisting dose-related effects. Psychopharmacology 218:649-665.

Griffiths RR, Johnson MW, Carducci MA, Umbricht A, Richards WA, Richards BD, Cosimano MP, Klinedinst MA (2016) Psilocybin produces substantial and sustained decreases in depression and anxiety in patients with life-threatening cancer: a randomized double-blind trial. J Psychopharmacol 30:1181-1197.

Griffiths RR, Johnson MW, Richards WA, Richards BD, Jesse R, MacLean KA, Barrett FS, Cosimano MP, Klinedinst MA (2018) Psilocybin-occasioned mystical-type experience in combination with meditation and other spiritual practices produces enduring positive changes in psychological functioning and in trait measures of prosocial attitudes and behaviors. J Psychopharmacol 32:49-69.

Holze F, Duthaler U, Vizeli P, Muller F, Borgwardt S, Liechti ME (2019) Pharmacokinetics and subjective effects of a novel oral LSD formulation in healthy subjects. Br J Clin Pharmacol 85:1474-1483.

Holze F, Vizeli P, Muller F, Ley L, Duerig R, Varghese N, Eckert A, Borgwardt S, Liechti ME (2020) Distinct acute effects of LSD, MDMA, and D-amphetamine in healthy subjects. Neuropsychopharmacology 45:462-471.

Holze F, Liechti ME, Hutten N, Mason NL, Dolder PC, Theunissen EL, Duthaler U, Feilding A, Ramaekers JG, Kuypers KPC (2021a) Pharmacokinetics and pharmacodynamics of lysergic acid diethylamide microdoses in healthy participants. Clin Pharmacol Ther 109:658-666.

Holze F, Vizeli P, Ley L, Muller F, Dolder P, Stocker M, Duthaler U, Varghese N, Eckert A, Borgwardt S, Liechti ME (2021b) Acute dose-dependent effects of lysergic acid diethylamide in a double-blind placebo-controlled study in healthy subjects. Neuropsychopharmacology 46:537-544.

Holze F, Ley L, Muller F, Becker AM, Straumann I, Vizeli P, Kuehne SS, Roder MA, Duthaler U, Kolaczynska K, Varghese N, Eckert A, Liechti ME (2022) Direct comparison of the acute effects of lysergic acid diethylamide and psilocybin in a double-blind placebo-controlled study in healthy subjects. Neuropsychopharmacology doi: 10.1038/s41386-022-01297-2.

Hysek CM, Liechti ME (2012) Effects of MDMA alone and after pretreatement with reboxetine, duloxetine, clonidine, carvedilol, and doxazosin on pupillary light reflex. Psychopharmacology (Berl) 224:363-376.

Hysek CM, Schmid Y, Simmler LD, Domes G, Heinrichs M, Eisenegger C, Preller KH, Quednow BB, Liechti ME (2014) MDMA enhances emotional empathy and prosocial behavior. Soc Cogn Affect Neurosci 9:1645-1652.

Janke W, Debus G (1978) Die Eigenschaftswörterliste. Göttingen: Hogrefe.

Liechti ME, Dolder PC, Schmid Y (2017) Alterations in conciousness and mystical-type experiences after acute LSD in humans. Psychopharmacology 234:1499-1510.

MacLean KA, Johnson MW, Griffiths RR (2011) Mystical experiences occasioned by the hallucinogen psilocybin lead to increases in the personality domain of openness. J Psychopharmacol 25:1453-1461.

Preller KH, Herdener M, Pokorny T, Planzer A, Kraehenmann R, Stämpfli P, Liechti ME, Seifritz E, Vollenweider FX (2017) The fabric of meaning and subjective effects in LSD-induced states depend on serotonin 2A receptor activation Curr Biol 27:451-457.

Roseman L, Nutt DJ, Carhart-Harris RL (2017) Quality of acute psychedelic experience predicts therapeutic efficacy of psilocybin for treatment-resistant depression. Front Pharmacol 8:974.

Ross S, Bossis A, Guss J, Agin-Liebes G, Malone T, Cohen B, Mennenga SE, Belser A, Kalliontzi K, Babb J, Su Z, Corby P, Schmidt BL (2016) Rapid and sustained symptom reduction following psilocybin treatment for anxiety and depression in patients with life-threatening cancer: a randomized controlled trial. J Psychopharmacol 30:1165-1180.

Schmid Y, Enzler F, Gasser P, Grouzmann E, Preller KH, Vollenweider FX, Brenneisen R, Mueller F, Borgwardt S, Liechti ME (2015) Acute effects of lysergic acid diethylamide in healthy subjects. Biol Psychiatry 78:544-553.

Studerus E, Gamma A, Vollenweider FX (2010) Psychometric evaluation of the altered states of consciousness rating scale (OAV). PLoS One 5:e12412.

Tagliazucchi E, Roseman L, Kaelen M, Orban C, Muthukumaraswamy SD, Murphy K, Laufs H, Leech R, McGonigle J, Crossley N, Bullmore E, Williams T, Bolstridge M, Feilding A, Nutt DJ, Carhart-Harris R (2016) Increased global functional connectivity correlates with LSD-induced ego dissolution. Curr Biol 26:1043-1050.
